# Novel Gradient p-Doping Strategy Enables Efficient Carbon-Based Hole Transport Layer-Free Perovskite Solar Cells

**DOI:** 10.1007/s40820-026-02112-z

**Published:** 2026-02-23

**Authors:** Junwei Xiang, Siqi Jiang, Yanjie Cheng, Weiting Du, Yuan Shi, Song Shen, Bolun Zhang, Qian Yue, Xinyi Xu, Anyi Mei, Yang Zhou, Yinhua Zhou, Hongwei Han

**Affiliations:** Michael Grätzel Center for Mesoscopic Solar Cells, Wuhan National Laboratory for Optoelectronics, Key Laboratory of Materials Chemistry for Energy Conversion and Storage of Ministry of Education, Huazhong University of Science and Technology, Wuhan, 430074 Hubei People’s Republic of China

**Keywords:** Printable mesoscopic perovskite solar cells, Gradient p-doping, Hole transport layer free, Carbon electrode, Charge carrier dynamics

## Abstract

Carbon-based hole transport layer-free (HTL-free) printable mesoscopic perovskite solar cells (p-MPSCs) are highly attractive for their low-cost and scalable fabrication. However, the intrinsically n-type nature of the perovskite, combined with the lack of an HTL, severely impedes hole extraction and limits device performance. In this work, we innovatively introduce a polymer with strong electron-withdrawing capability as an additive into p-MPSCs. Owing to its large molecular size, this polymer spontaneously forms a negative gradient distribution from top to bottom within the mesoporous scaffold during fabrication. This distribution creates a favorable gradient p-doping profile within p-MPSCs, which facilitates more efficient hole transport, a finding corroborated by combined device simulation and cross-sectional photoluminescence mapping. Consequently, the optimized p-MPSCs exhibit an average open-circuit voltage enhancement of over 50 mV, a steady-state power conversion efficiency of 21.56% and operational stability exceeding 1500 h at 55 °C under simulated 1-sun illumination using a halogen lamp without a UV filter.

**Supplementary Information:**

The online version contains supplementary material available at 10.1007/s40820-026-02112-z.

## Highlights

- A novel strategy of creating spontaneous formation of gradient p-doping in perovskite embedded in mesoporous oxide scaffold is developed.
- The feasibility of implementing a gradient p-doping strategy in printable mesoscopic perovskite solar cells is demonstrated for the first time through combined device simulations and cross-sectional photoluminescence mapping.
- The resulting carbon-based hole transport layer-free solar cell exhibits outstanding power conversion efficiency along with superior operational stability over 1500 h without UV filter at 55 °C.

**Supplementary Information:**

The online version contains supplementary material available at 10.1007/s40820-026-02112-z.

## Graphical abstract

**Figure:**
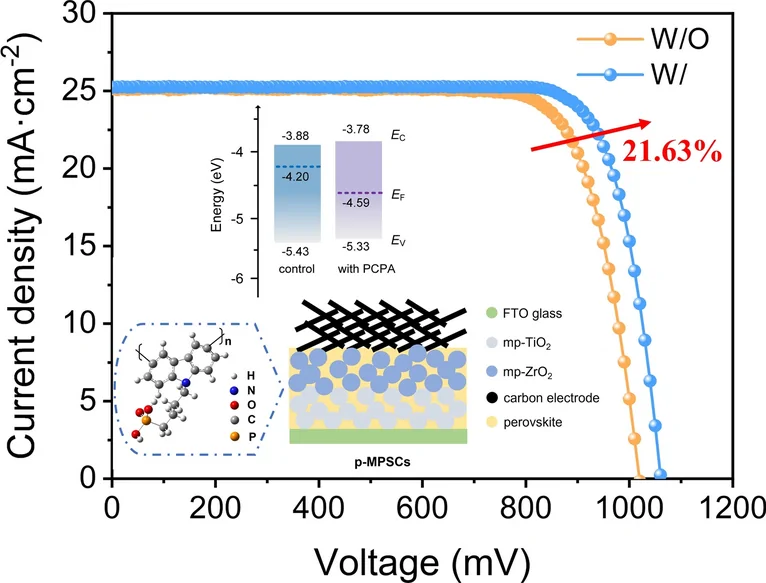

## Introduction

As the need to combat climate change and adopt renewable energy intensifies, solar energy has become a critical pillar of modern clean energy strategies. Among emerging photovoltaic (PV) technologies, metal halide perovskite solar cells (PSCs) have quickly gained prominence thanks to their remarkable power conversion efficiencies (PCE) obtained through low-temperature and low-cost fabrication [1–4]. However, the conventional PSCs with p-i-n or n-i-p structures inevitable require the inert atmosphere or vacuum for the deposition of the perovskite or metal electrode, which limits their scalability [5–11]. In contrast, printable mesoscopic perovskite solar cells (p-MPSCs) effectively overcome these limitations. During its fabrication, a triple-layer scaffold of mesoporous TiO_2_ (mp-TiO_2_)/mesoporous ZrO_2_ (mp-ZrO_2_)/porous carbon, is firstly screen-printed onto the substrate, followed by drop-casting of the perovskite precursor solution into it [12]. The entire process is carried out in ambient air and highly compatible with large-scale production [13, 14]. The substitution of noble metal by low-cost carbon for electrode, along with the elimination of expensive hole transport layer (HTL), further reduces manufacturing costs. These make p-MPSC promising for next-generation solar energy harvesting.

However, the distinctive architecture of p-MPSCs introduces more substantial energy losses compared to conventional PSCs [15–17]. In these devices, photogenerated charge carriers are principally produced in the perovskite infiltrated within the mp-TiO_2_ layer with a thickness of ~ 0.8 μm [18, 19], where electrons can be rapidly extracted by the adjacent TiO_2_. Due to the resolution limits of the screen-printing technique, the mp-ZrO_2_ layer must be at least ~ 2 μm thick to effectively insulate the two adjacent layers [20]. In this way, photogenerated holes must diffuse over an additional 2 μm perovskite in the mp-ZrO_2_ layer to reach the carbon electrode. Given that perovskite materials are inherently defective and typically exhibit n-doping characteristics, this extended hole transport pathway significantly compromises charge collection efficiency and overall device performance [21–23]. Consequently, mitigating n-doping effects and reducing defect densities in the perovskite absorber are critical for achieving high-performance p-MPSCs.

Several strategies have been developed to mitigate n-doping in perovskite materials. One approach involves modulating the perovskite stoichiometry to control doping characteristics. For instance, reducing the PbX_2_/AX ratio (where X = I or Br, and A = Cs, MA, or FA) can shift the perovskite from *n*-type to weakly *p*-type doping [24]. However, this method shows limited effectiveness as significant deviation from stoichiometric composition often compromises superior optoelectronic properties of the perovskite. Metal dopants, such as Sn, Bi, Ag, Na, and Rb, have also been shown to induce p-doping in perovskites [25–29]. However, these approaches typically require high doping concentrations or introduce additional trap states, both of which adversely affect charge carrier transport in the perovskite material [30–33]. Charge transfer doping has emerged as an effective strategy for modulating perovskite doping characteristics. This process involves carrier exchange between the perovskite and an external molecular species at surfaces, grain boundaries, or interfaces, without the need to incorporate the species into the crystal lattice. Through interfacial electron transfer, this mechanism induces Fermi-level realignment, alters free-carrier concentrations, and generates band bending [34–37]. Recently, Xiong et al. reported that 4PACz, a small molecular carbazole phosphonic acid dopant, can successfully convert perovskite from *n*-type to *p*-type doping by charge transfer doping, due to its strong electron-withdrawing nature. The phosphonic acid group in 4PACz also forms strong bonding with uncoordinated Pb^2+^, inhibiting the formation of the *n*-type halide defects. This approach has been particularly effective in perovskite light emitting diodes, leading to significant device performance enhancement [38]. This p-doping strategy also works in PSCs with carbon electrode. For example, Du et al. systematically demonstrated that p-doping of the carbon/HTL interface significantly reduces the hole extraction barrier. Further Drift–diffusion simulations revealed that such interfacial p-doping suppresses non-radiative recombination and enhances quasi-Fermi-level splitting by forming a beneficial energy level alignment at the back contact [39]. These findings underscore p-doping as a universal and effective strategy for modifying the electronic properties of perovskite materials and enhancing device performance.

In p-MPSCs, achieving optimal device performance expects distinct doping profiles across the mesoporous scaffold. The perovskite infiltrating the mp-TiO_2_ layer should remain intrinsically neutral, while the perovskite within the mp-ZrO_2_ layer requires controlled *p*-type doping to enable efficient charge separation and directional carrier extraction. However, conventional small-molecule dopants typically infiltrate uniformly throughout the perovskite during deposition, which possibly lead to homogeneous doping. This non-selective doping profile could fail to meet the desired spatial doping requirements and consequently limit efficiency improvements in p-MPSCs.

In this work, a polymer dopant, poly-[(4-(9H-carbazol-9-yl) butyl)] phosphonic acid (PCPA) was introduced into p-MPSCs to spatially modulate the doping of the perovskite within the mesoporous scaffold. PCPA exhibits a negative concentration gradient from the top mp-ZrO_2_ toward the bottom mp-TiO_2_, which is spontaneously formed due to its relatively large size compared with the mesopores. The strong electron-accepting character of PCPA effectively counteracts the intrinsic n-doping of perovskite. In the mp-TiO_2_ layer, where PCPA concentration is minimal, the doping modification remains negligible, preserving unimpaired electron extraction. Conversely, in the mp-ZrO_2_ layer, the increasing PCPA concentration creates a beneficial p-doping gradient. Furthermore, PCPA passivates defects through bonding between its phosphonic acid groups and undercoordinated Pb^2+^ sites. These synergistic effects collectively enhance charge transport while suppressing recombination, boosting the device PCE from 20.05% to 21.63%. The PCPA-treated device also exhibits remarkable operational stability, maintaining 90% of its initial efficiency after 1500 h operation at maximum power point (MPP) under a halogen lamp without UV filter and with intensity calibrated to 1-sun at (55 ± 5) °C.

## Experimental Section

### Materials

Lead (II) iodide (PbI_2_, 99.99%) was supplied by TCI Co. Ltd. Formamidinium iodide (FAI, > 99.99%), methylammonium iodide (MAI, > 99.99%), methylammonium chloride (MACl, 99.99%), and nanoparticulate titanium dioxide paste (TiO_2_, NR30) were obtained from GreatCell Solar. Cesium iodide (CsI, > 99.9%) was sourced from Xi’an Polymer Light Technology Corp. All solvents, including N, N-dimethylformamide (DMF, 99.8%) and dimethyl sulfoxide (DMSO, 99.7%) were purchased from Sigma-Aldrich. Titanium diisopropoxide bis(acetylacetonate) (75 wt% in isopropanol), ethyl cellulose (viscosity grades 10cP and 46cP), and terpineol were also purchased from Sigma-Aldrich. PCPA (Mw 4000–6000) was provided by Weiran Materials. Fluorine-doped tin oxide (FTO) glass substrates were acquired from Yingkou OPV Tech Co., Ltd. The ZrO_2_ and carbon pastes were sourced from WonderSolar Co., Ltd. All chemical reagents and materials used in this study were utilized without further purification.

### Precursor Preparation

The perovskite precursor solution was prepared by dissolving MACl (0.0135 g, 0.2 mmol), CsI (0.0130 g, 0.05 mmol), MAI (0.0239 g, 0.15 mmol), FAI (0.1376 g, 0.8 mmol), and PbI_2_ (0.4610 g, 1 mmol) in 0.8 mL of a mixed solvent composed of DMF and DMSO (volume ratio 4:1). Then, PCPA was added to the mixture with prespecified concentration, and the solution was stirred at 55 °C for 1 h for well dispersion.

### Device Fabrication

The fabrication process began with laser etching of the FTO glass to define the electrode pattern, followed by ultrasonic cleaning in detergent, deionized water, and ethanol for 10 min each. A compact TiO_2_ layer was then deposited onto the cleaned FTO via spray pyrolysis using a titanium diisopropoxide bis(acetylacetonate) precursor at 450 °C. Subsequently, a mesoporous TiO_2_ layer was screen-printed and annealed at 500 °C for 40 min. The mesoporous ZrO_2_ and porous carbon layers were sequentially screen-printed on top and annealed at 400 °C for 40 min to form the triple-layer mesoporous scaffold. After cooling to room temperature, the perovskite precursor solution was infiltrated into the scaffold and annealed at 56 °C for 18 h to complete the device. To achieve optimal performance, the annealing step was conducted under ambient air conditions in a fume hood at a temperature of (20 ± 3) °C with a relative humidity of (30 ± 10)%.

### Computational Method

The simulations of carbon-based HTL-free PSCs were conducted using the SCAPS-1D program under standard AM 1.5G illumination, with impedance losses neglected to isolate the intrinsic device behavior. The simulated device architecture was configured as FTO/TiO_2_/perovskite/carbon. In p-MPSCs, thicknesses of the mp-TiO_2_ and mp-ZrO_2_ layers are approximately 800 and 2000 nm, respectively. Perovskite embedded in the mp-TiO_2_ layer functions mainly as the light absorber, whereas perovskite within the mp-ZrO_2_ layer serves solely for transporting photogenerated carriers. To accurately simulate the two functional regions within the p‑MPSC device architecture, the perovskite layer in the model is divided into an 800 nm‑thick illuminated and a 2000-nm‑thick dark region, representing the perovskite in the mp‑TiO_2_ layer and mp‑ZrO_2_ layer, respectively. In the simulation, photogenerated carriers were restricted to be produced in the light-absorbing region, while the dark region functioned solely as a transport pathway for holes toward the carbon counter electrode. To evaluate the impact of doping on device performance, the baseline perovskite was modeled as an intrinsitic weakly n-type semiconductor with a doping concentration of 1 × 10^14^ cm^−3^. For the gradient p-doping scenario, the light-absorbing region maintained the same *n*-type characteristics, whereas the dark region was assigned a gradient *p*-type doping profile ranging from 0 to 1 × 10^17^ cm^−3^, increasing linearly from the interface with the light-absorbing layer toward the carbon electrode. Detailed parameters used in the simulation are summarized in Tables S1 and S2.

## Results and Discussion

### Interactions Between PCPA and Perovskite

**Fig. 1:**
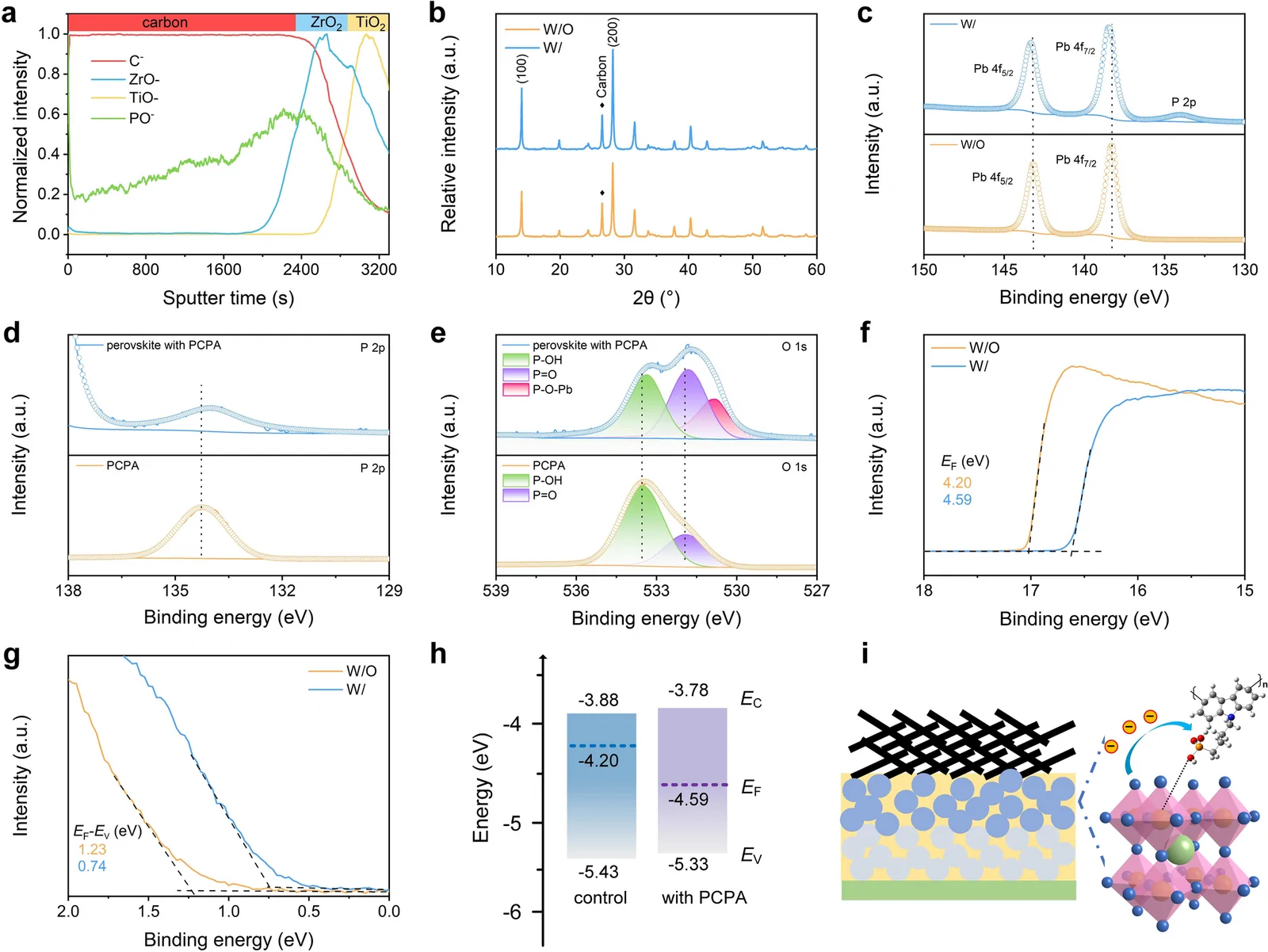
**a** TOF-SIMS depth profiles of the p-MPSC with the addition of PCPA. **b** XRD patterns of p-MPSCs without and with PCPA. **c** XPS Pb 4*f* spectra of perovskite films without and with PCPA. **d** XPS P 2*p* spectra of PCPA powder and the mixture of PCPA and perovskite. **e** XPS O 1*s* spectra of perovskite film with PCPA and pure PCPA powder. **f** Secondary-electron cutoff and **g** valence band edges from UPS spectra of perovskite films without and with PCPA. **h** Energy level diagrams of perovskite films without and with PCPA. **i** Schematic of PCPA induced charge transfer doping in p-MPSCs

Figure S1a depicts the screen-printing process employed in the fabrication of p-MPSCs. In this method, the paste was applied onto a mesh screen positioned above the substrate. Using a squeegee to sweep across the screen, layers of TiO_2_, ZrO_2_, and carbon pastes were sequentially printed. After deposition, the printed layers were thermally annealed to eliminate organic pore-forming agents, yielding a mp-TiO_2_/mp-ZrO_2_/porous carbon scaffold. The perovskite precursor solution was then drop-cast to infiltrate the scaffold, followed by a final annealing step to complete the p-MPSC device (Fig. S1b). The PCPA was introduced into the device by adding it to the perovskite precursor solution as an additive, and its molecular structure is shown in Fig. S2. We first employed time-of-flight secondary ion mass spectrometry (TOF-SIMS) to analyze the spatial distribution of PCPA in the p-MPSC. Depth profiles of C^−^, ZrO^−^, and TiO^−^ fragments demonstrate the distinct triple-layer structure of TiO_2_/ZrO_2_/carbon (Fig. 1a). By tracking the signal of the PO^−^ fragment, which originates exclusively from the phosphonic acid group in PCPA, it is shown that PCPA accumulates at the carbon/mp-ZrO_2_ interface and decreases monotonically in concentration toward the interior of the mp-TiO_2_ layers. This can be possibly attributed to the relatively large molecular size of PCPA compared to the size of the mesopores, which hinders its further infiltration into the mp-TiO_2_ [40].

Both p-MPSCs without and with PCPA demonstrate compact filling of perovskite inside the mesoporous oxide scaffold as evidenced by the cross-sectional scanning electron microscope (SEM) (Fig. S3). The influence of PCPA on perovskite crystallinity in the p-MPSC was investigated by X-ray diffraction (XRD) analysis. For both devices, distinct peaks at 13.96° and 28.13° are observed and are attributed to the (100) and (200) crystal planes of the perovskite, respectively (Fig. 1b). Besides, enhancements in the intensity of these diffraction peaks are observed upon introducing PCPA, suggesting that its presence improves perovskite crystallinity within the mesoporous film. The possible interactions between PCPA and the perovskite were investigated by X-ray photoelectron spectroscopy (XPS), and full XPS spectra of perovskite films without and with PCPA, and pure PCPA powder are shown in Fig. S4. As shown in Fig. 1c, the pristine perovskite film exhibits Pb 4*f*_5/2_ and Pb 4*f*_7/2_ peaks at 143.19 and 138.28 eV, respectively. Upon the incorporation of PCPA, both peaks shift to higher binding energies of 143.34 and 138.45 eV. This indicates a reduction in electron density surrounding Pb^2+^, suggesting the electron-withdrawing ability of PCPA. Additionally, a new peak emerges at 134.00 eV in the sample with PCPA, which corresponds to the P 2*p* orbital, thereby confirming the successful incorporation of phosphorus-containing PCPA into the perovskite film following annealing. Figure 1d further compares the P 2*p* spectra of pure PCPA powder and PCPA-contained perovskite films. By incorporating into the perovskite, the P 2*p* binding energy decreases from 134.23 to 134.00 eV, indicating increased electron density surrounding phosphorus atoms. This result further corroborates the electron-withdrawing nature of PCPA upon interaction with the perovskite material. In addition, O 1*s* spectra provide further evidence of how PCPA interacts with the perovskite (Fig. 1e). In the O 1*s* spectrum of PCPA powder, two distinct peaks at 533.53 and 531.92 eV are observed, corresponding to O from P-OH and P=O groups, respectively. Once PCPA incorporated into the perovskite, a new peak at 530.81 eV emerges in the O 1*s* spectrum, which is possibly caused by the formation of P–O–Pb bonding [38]. This indicates that P–OH groups can deprotonate and bond with undercoordinated Pb^2+^, thereby bridging PCPA and perovskite to enable electron transfer from perovskite to PCPA.

Ultraviolet–visible (UV–vis) spectroscopy reveals that the addition of PCPA barely changes the perovskite absorption (Fig. S5a), while Tauc plot analysis shows that the same optical bandgap of 1.55 eV for both perovskite thin films without and with PCPA (Fig. S5b). As PCPA demonstrates electron-withdrawing feature, its addition could change the energy levels in the perovskite [21, 38]. Therefore, we conducted ultraviolet photoelectron spectroscopy (UPS) measurements to elucidate this. The spectra near the secondary electron cutoff (SEC) and the valence band maximum (VBM) are shown in Fig. 1f, g, respectively, for perovskite thin films without and with PCPA. Based on the SEC, VBM spectrum, and the bandgap, positions of Fermi level (*E*_F_), VBM, and conduction band minimum (CBM) can be obtained as shown in Fig. 1h. The pristine perovskite is n-type doped as its *E*_F_ is adjacent to CBM (Fig. 1h). With the incorporation of PCPA, the *E*_F_ downshifts toward VBM and converts the perovskite from n-doping to weak p-doping (Fig. 1i). In addition, the change in work function for perovskite films without and with PCPA was further verified by Kelvin probe measurements (Fig. S6). It should be noted that these measurements were conducted under ambient conditions, which may introduce slight deviations from the UPS results. Nevertheless, the overall trend consistently indicates an increased work function and reduced n-type doping in the PCPA-treated perovskite film. Such a change in perovskite doping characteristic could alter the transportation of charge carriers within p-MPSC and affect its performance.

### Device Simulation for Gradient p-Doping (GPD) Strategy

Based on the above-mentioned analysis, the introduction of PCPA can cause gradient p-doping (GPD) in the p-MPSC. We further simulated how the modulation of doping in perovskite affects the performance of carbon-based HTL-free PSCs by using SCAPS-1D software (version 3.3.10) [41]. The device architecture employed for simulation is fluorine-doped tin oxide (FTO)/electron transport layer (ETL)/perovskite/carbon. The total thickness of the perovskite absorber is 2.8 μm and is divided into 2 distinct functional regions. The first 2 μm perovskite adjacent to carbon operates primarily as a charge-transport region under dark, while the rest 0.8 μm perovskite serves as the photoactive region, responsible mainly for photocarrier generation. The key parameters used in the simulations were carefully selected based on well-established literature references [42–45] and are provided in Tables S1 and S2. In the pristine device, the perovskite absorber was modeled to be weakly n-type doped to account for its intrinsic n-doping characteristic [46, 47]. For the doped variant, a spatially GPD profile was implemented across the 2 μm dark region of the perovskite, with concentration decreasing gradually from the carbon/perovskite interface.

**Fig. 2:**
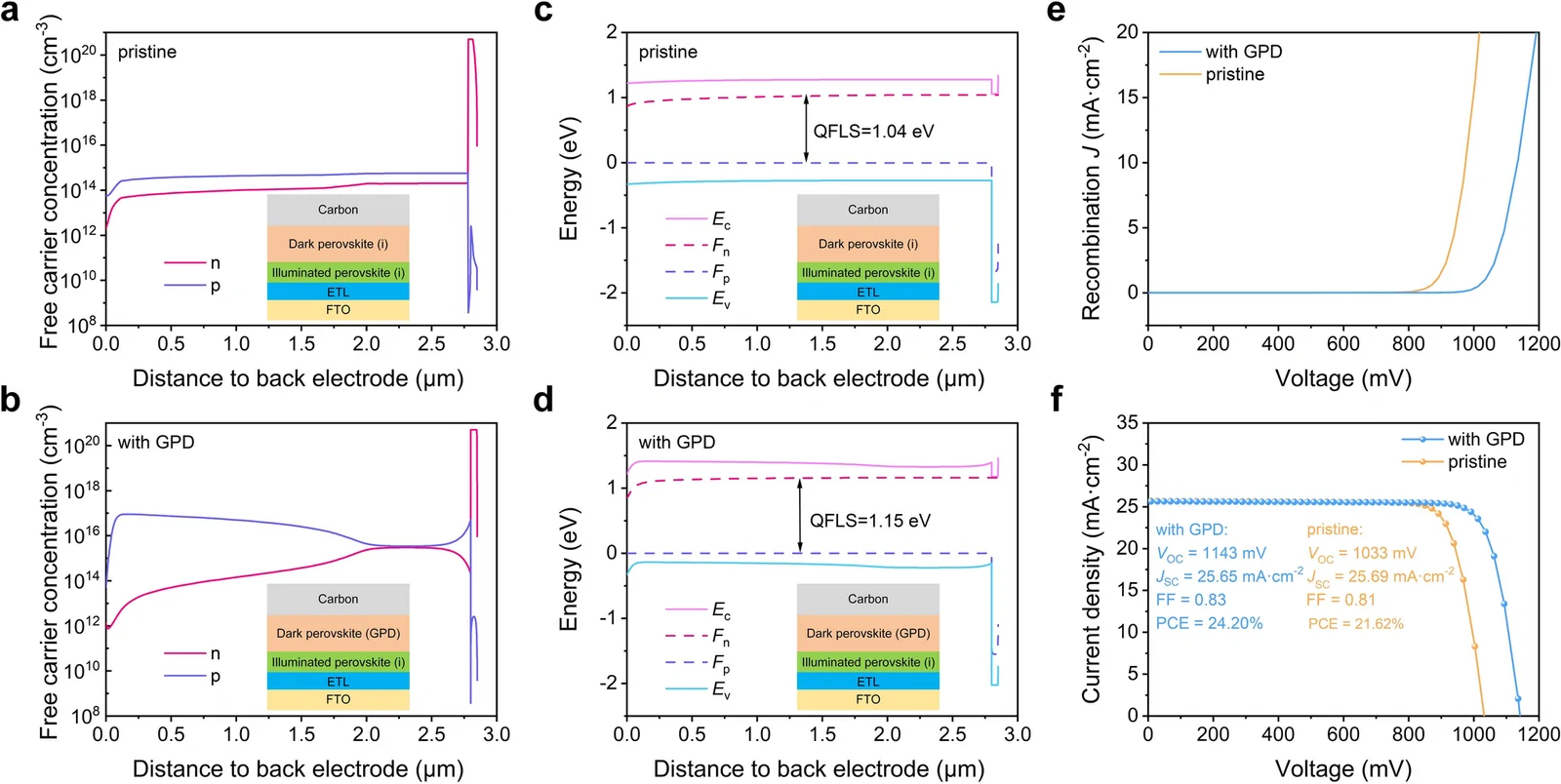
Simulated energy levels across carbon-based HTL-free PSCs based on **a** pristine and **b** with GPD perovskites. Distributions of free-carrier concentrations across carbon-based HTL PSCs based on **c** pristine and **d** with GPD perovskites. **e** Recombination current density at the back contact of carbon-based HTL-free PSCs based on pristine and with GPD perovskites. **f** Simulated PCE of carbon-based HTL-free PSCs based on pristine and with GPD perovskites

Figure 2a, b presents the simulated spatial concentration distributions of photogenerated charge carriers across the perovskite layer in the pristine and GPD devices under illumination. In the pristine device, the electron concentration in the illuminated region is approximately one order of magnitude lower than the hole concentration, primarily due to efficient electron extraction at the ETL/perovskite interface (Fig. 2a). This concentration disparity remains relatively unchanged in the dark region, as the absence of a HTL limits further charge carrier separation. In contrast, the perovskite in the GPD device exhibits identical electron and hole concentrations in the illuminated region. However, upon extending into the dark region toward the carbon electrode, the hole concentration gradually increases while the electron concentration decreases (Fig. 2b). This behavior arises from the introduction of GPD in the dark region, which enhances charge separation across the whole device. The enhanced charge carrier separation by the introduction of GPD increases the quasi-Fermi-level splitting (QFLS) (Fig. 2c, d) and suppresses the unwanted charge carrier recombination at the back contact (Fig. 2e). As a result, the open-circuit voltage (*V*_OC_) loss was reduced greatly, which primarily leads to the improvement of device PCE from 21.62% to 24.20% (Fig. 2f) [48].

### Characterization of Carrier Dynamics

**Fig. 3:**
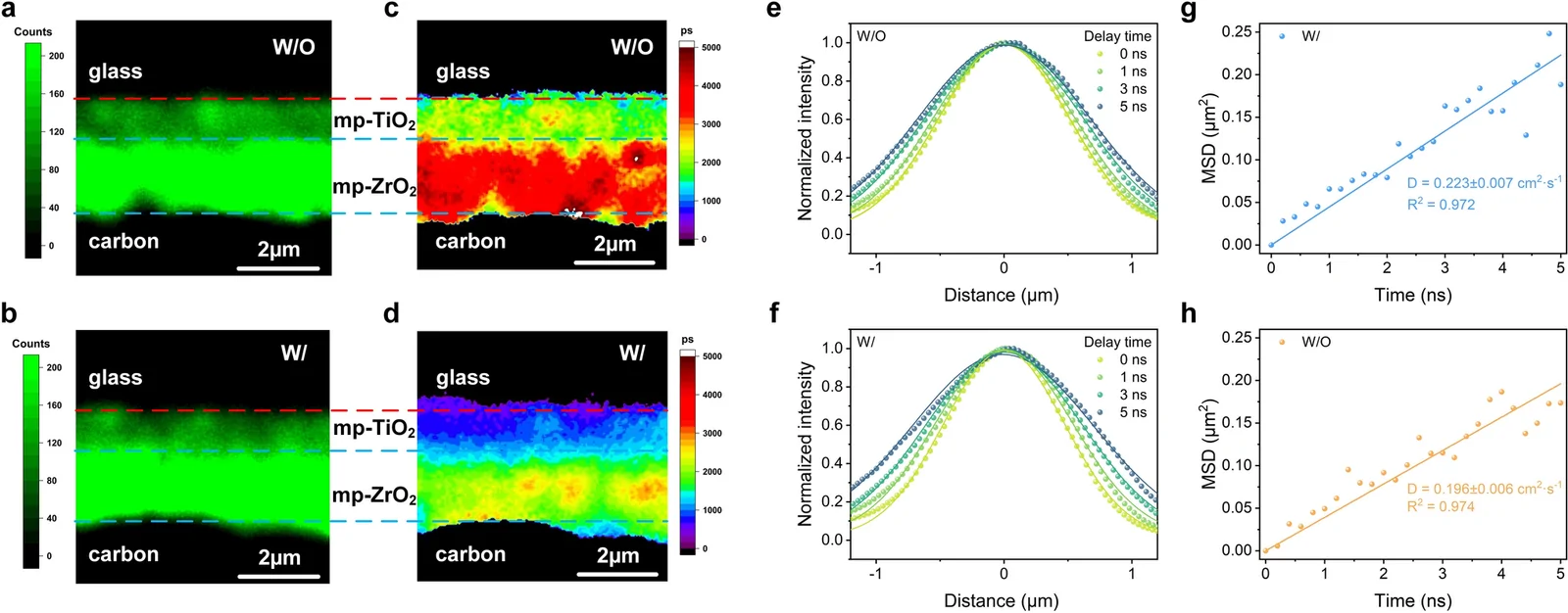
PL intensity mapping of cross-sectional p-MPSCs **a** without **b** with PCPA. PL lifetime mapping of cross-sectional p-MPSCs **c** without **d** with PCPA. Normalized 1D PL intensity distributions extracted from temporal series of PL spatial profiles **e** without **f** with PCPA. Linearly fittings of carriers’ MSD as a function of time **g** without **h** with PCPA

The effect of PCPA doping on charge carrier dynamics in p-MPSCs was further investigated using photoluminescence (PL) intensity and lifetime imaging on cross-sectional devices. PL intensity and the corresponding lifetime distribution images were obtained from the same position, allowing for direct correlation between emission intensity and carrier lifetime. For both samples without and with PCPA, the perovskite in the mp-TiO_2_ layer exhibited lower PL intensity and shorter carrier lifetime compared to that in the mp-ZrO_2_ layer (Fig. 3a–d). This phenomenon is attributed to strong electron extraction effect imposed by TiO_2_ [49]. Upon introducing PCPA, a noticeable reduction in overall carrier lifetime was observed in the device (Fig. 3c, d). This decrease is likely due to enhanced hole extraction enabled by the GPD in the perovskite produced by the gradient distribution of PCPA [50].

The carrier transportation behavior within the perovskite embedded in mp-ZrO_2_ layer was further explored by the PL-based carrier diffusion test. During the test, the pulsed laser beam was fixed at the cross-sectional center of the mp-ZrO_2_ layer (Fig. S7), while the detector continuously recorded the temporal evolution of the PL spatial profile [51]. The temporal spreading of the PL spatial profile is observed for p-MPSCs both without and with PCPA (Fig. S8). To quantify the diffusion process, one-dimensional PL intensity profiles were extracted along the vertical axis of maximum intensity in each image and were fitted by using Gaussian functions (Fig. 3e, f). These Gaussian fits were then used to compute the temporal evolution of the mean squared displacement (MSD) of charge carriers, and the linear fitting of this series of MSD can be then used to calculate the carrier diffusion constant (Fig. 3g, h) [52–54]. Upon the addition of PCPA, the diffusion constant of carriers in the perovskite filled in mp-ZrO_2_ increased from 0.196 to 0.223 cm^2^ s^−1^ (Fig. 3g, h), indicating improved carrier mobility. This phenomenon can be attributed to two primary factors. First, PCPA passivates uncoordinated Pb^2+^ defects that serve as non-radiative recombination centers (Fig. 1c–e). Second, PCPA modifies the doping profile by transitioning the perovskite from strong *n*-type to weak *p*-type doping (Fig. 1h). This adjustment balances electron and hole concentrations, thereby enhancing the probability of radiative recombination during carrier diffusion.

Furthermore, the Nyquist plot obtained by electrochemical impedance spectroscopy (EIS) was employed to analyze the charge transport and recombination processes in the devices (Fig. S9). In the Nyquist plots, the high-frequency arc is associated with interfacial charge transfer resistance, while the low-frequency arc reflects charge recombination behavior. Upon introducing PCPA, the high-frequency semicircle becomes smaller, indicating reduced charge transfer resistance, whereas the low-frequency semicircle expands, suggesting suppressed carrier recombination [55]. These results corroborate that the GPD strategy substantially improves charge extraction and transport across the p-MPSCs.

### Characterization of Defect Passivation

**Fig. 4:**
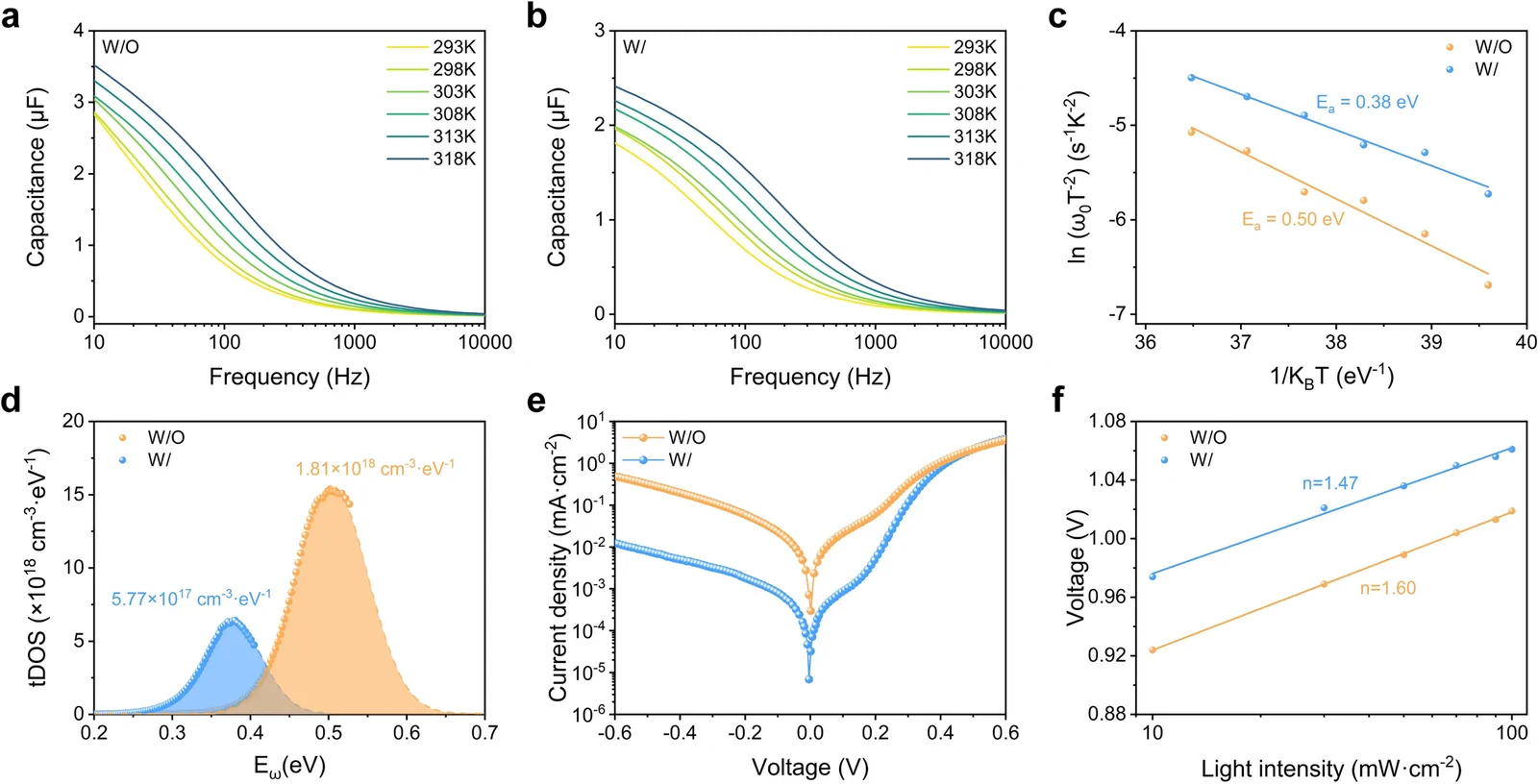
Temperature dependent C-F plots of p-MPSCs **a** without and **b** with PCPA. **c** Arrhenius plots of the characteristic frequencies at different temperatures, **d** tDOSs, **e** dark *J*-*V* curves and **f** light intensity dependences of *V*_OC_ of p-MPSCs without and with PCPA

Thermal admittance spectroscopy (TAS) was then used to analyze the impact of PCPA incorporation on trap states in p-MPSCs. For both devices without and with PCPA, the capacitance-frequency (C-F) curves shift toward higher frequencies as the temperature increases (Figs. 4a, b and S10), indicating temperature-dependent carrier de-trapping process [56]. From these data, the defect activation energy (*E*_a_), which represents the energy required for charge carrier de-trapping from a defect, was extracted and is presented in Fig. 4c. The *E*_a_ value decreases from 0.50 to 0.38 eV by PCPA modification, implying an increased probability of defect de-trapping [57–60]. Additionally, by utilizing the C–F curves at varied temperatures, the trap density of states (tDOS) in p-MPSCs could be derived [56, 58]. It is determined that the tDOS is markedly reduced from 1.81 × 10^18^ to 5.77 × 10^17^ cm^−3^ eV^−1^ after PCPA incorporation (Fig. 4d), further confirming the dopant’s effectiveness in passivating defects. Dark current density–voltage (*J*-*V*) measurements (Fig. 4e) also indicate a reduction in leakage current for the PCPA-treated device, suggesting decreased non-radiative recombination induced charge carrier loss. The influence of PCPA on recombination processes was further evaluated by analyzing the dependence of *V*_OC_ on light intensity (Fig. 4f). The ideality factor n is reduced from 1.60 to 1.47 after doping, indicating a decrease in defect-assisted recombination [61]. Moreover, Mott–Schottky analysis (Fig. S11) reveals an increase in built-in potential (*V*_bi_) from 0.95 to 0.99 V for the device containing PCPA, which benefits charge separation and extraction. This is further confirmed by a shortened transient photocurrent (TPC) decay time from 8.18 to 5.14 µs (Fig. S12 and Table S3). Collectively, the above-mentioned findings confirm that the addition of PCPA effectively reduces defect densities and suppresses recombination losses, which would ultimately lead to improved performance in p-MPSCs.

### Device Performance

**Fig. 5:**
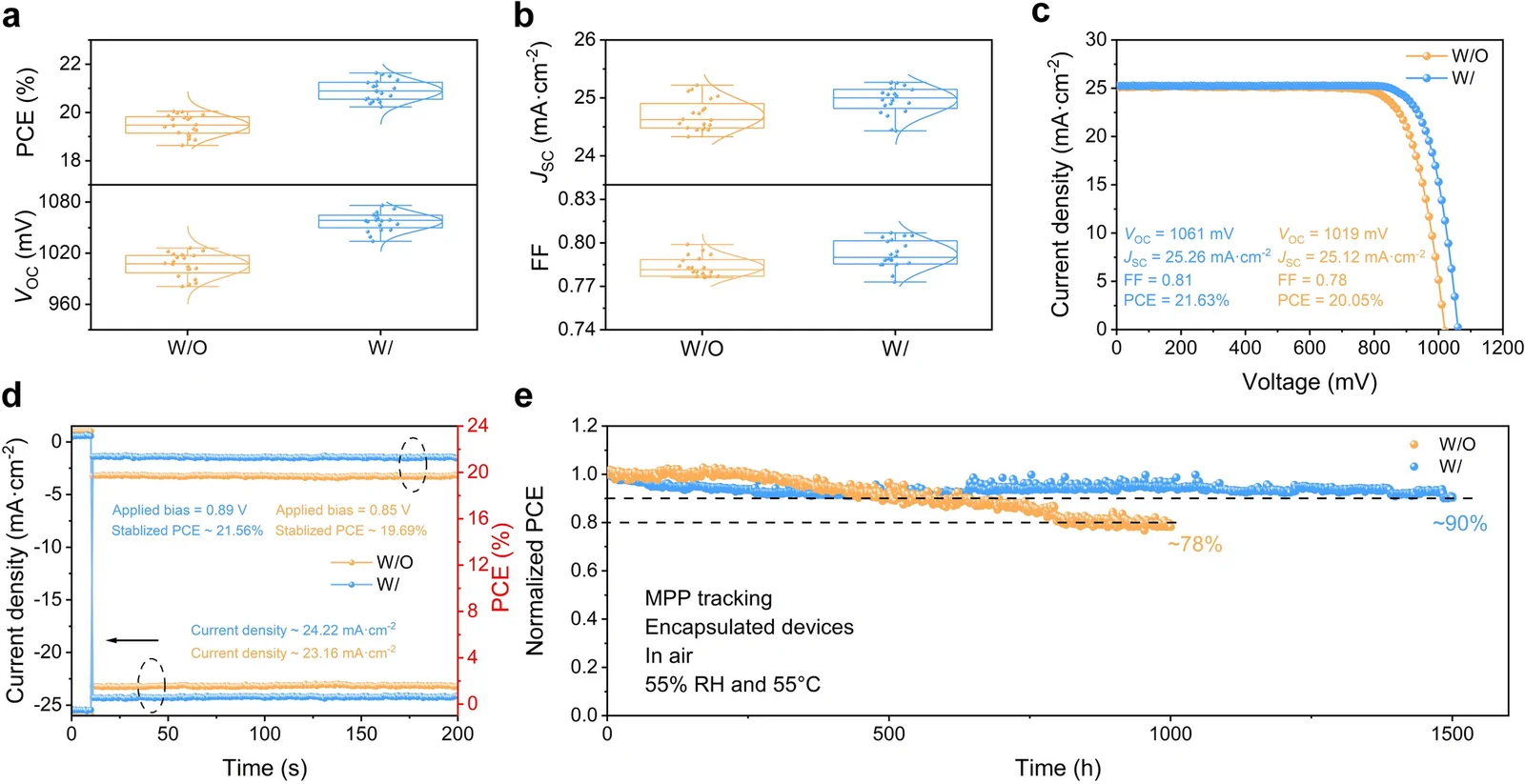
Statistics of **a** PCE and *V*_OC_, and **b** *J*_SC_ and FF of p-MPSCs without and with PCPA. **c** *J*-*V* curves of champion p-MPSCs without and with PCPA (20 devices per group). **d** Steady-state power output of p-MPSCs without and with PCPA over 200 s at MPP. **e** MPPT of encapsulated p-MPSC with PCPA under 100 mW cm^−2^ halogen lamp illumination

The PV performance of p-MPSCs was evaluated through *J-V* measurements. A series of devices were fabricated with PCPA concentrations ranging from 0 to 5 mg mL^−1^, among which 1.5 mg mL^−1^ was identified as optimal based on statistical analysis of the PV parameters (Fig. S13 and Table S4). Statistics of PV parameters of p-MPSCs without and with the addition of 1.5 mg mL^−1^ PCPA are given in Fig. 5a, b. Upon PCPA addition, averages of *V*_OC_, short-circuit current density (*J*_SC_), fill factor (FF) and PCE are improved from (1005.90 ± 13.54) mV, (24.71 ± 0.26) mA cm^−2^, (0.783 ± 0.0070), and (19.47 ± 0.41)% to (1057.10 ± 10.36) mV, (24.96 ± 0.24) mA cm^−2^, (0.792 ± 0.009), and (20.91 ± 0.42)%, respectively. These improvements can be largely attributed to the GPD induced by the addition of PCPA within p-MPSC, which increases hole transport and extraction efficiency while simultaneously reducing trap-assisted recombination by lowering defect densities (Figs. 2 and 3). The PCE, *V*_OC_, *J*_SC,_ and FF of the best performing device are improved by PCPA incorporation, from 20.05%, 1019 mV, 25.12 mA cm^−2^, and 0.78% to 21.63%, 1061 mV, 25.26 mA cm^−2^, and 0.81, respectively (Fig. 5c). In addition, analysis of the FF improvement indicates that the GPD strategy effectively reduces both non-radiative recombination losses and transport losses (Fig. S14). This facilitates more efficient carrier transport within the p-MPSCs and ultimately enhances the overall device performance [62]. Forward *J*-*V* scans of best performing devices without and with PCPA are shown in Fig. S15. These scans were employed for hysteresis analysis, which reveals a reduction in the hysteresis index from 4.64 to 4.02% by PCPA modification (Table S5). This improvement is possibly due to accelerated hole extraction, leading to a more balanced charge extraction between holes and electrons in the p-MPSCs after the incorporation of PCPA [63–65]. The steady-state power outputs of devices without and with PCPA were measured at a fixed bias of *V*_MPP_ and are shown in Fig. 5d. After being continuously tracked at 0.85 V for 200 s, the device without PCPA stabilizes at a current density of 23.16 mA cm^−2^ and a PCE of 19.69%, while the device with PCPA reaches a current density of 24.22 mA cm^−2^ and a PCE of 21.56% at a biased voltage of 0.89 V. These results consistently affirm the performance enhancement achieved by PCPA incorporation and demonstrate marginal deviations from PCEs obtained by *J-V* scans (Fig. 5c). Figure S16 shows the incident photon-to-current conversion efficiency (IPCE) spectra. The integrated *J*_SC_ values obtained from the IPCE spectra are 23.97 and 24.28 mA cm^−2^ for the devices without and with PCPA, respectively, consistent within the *J*_SC_ statistics obtained by *J-V* measurements. Finally, the long-term operational stability of the devices was assessed by maximum power point tracking (MPPT) under simulated 1-sun illumination. Devices were encapsulated and were placed under ambient air conditioning with a controlled relative humidity of (55 ± 5)% at (55 ± 5) °C during the test. Illumination was provided by a halogen lamp at an intensity of 100 mW⋅cm^−2^, without the use of a UV filter. As shown in Fig. 5e, the p-MPSC with PCPA demonstrates excellent operational stability, which preserves 90% of its initial PCE after 1500 h.

## Conclusion

In this study, a polymer-based p-type dopant of PCPA was introduced into p-MPSCs to enhance the performance. Owing to its large molecular size relative to the nanoscale mesopores, PCPA predominantly accumulates at the carbon/mp-ZrO_2_ interface and gradually decreases in concentration toward the interior of the mesoporous scaffold. This unique gradient distribution of PCPA, combined with its strong electron-withdrawing feature, induces effective GPD within the device, facilitating more efficient hole extraction and transport. Moreover, PCPA could bond with undercoordinated Pb^2+^ and effectively passivates charge carrier traps within the device. This establishes a robust electrical connection while suppressing non-radiative recombination. As a result, the average *V*_OC_ loss is reduced significantly by 50 mV, which contributes largely to the steady-state PCE improvement from 19.69% to 21.56%. Besides, the PCPA-treated device also exhibits remarkable operational stability, maintaining 90% of its initial efficiency after 1500 h MPPT under a halogen lamp without UV filter and with intensity calibrated to 1-sun at (55 ± 5) °C.

## Supplementary Information

Below is the link to the electronic supplementary material.

Supplementary file1 (DOCX 19931 KB)

## References

[1] A.K. Jena, A. Kulkarni, T. Miyasaka, Halide perovskite photovoltaics: background, status, and future prospects. Chem. Rev. **119**(5), 3036–3103 (2019). 10.1021/acs.chemrev.8b00539 30821144 10.1021/acs.chemrev.8b00539

[2] J.Y. Kim, J.-W. Lee, H.S. Jung, H. Shin, N.-G. Park, High-efficiency perovskite solar cells. Chem. Rev. **120**(15), 7867–7918 (2020). 10.1021/acs.chemrev.0c00107 32786671 10.1021/acs.chemrev.0c00107

[3] T. Du, H.U. Dag, Z. Peng, J. Englhard, A. Barabash et al., Enhancing the viability of p-i-n perovskite solar cells with printable carbon cathode: origin of polarity inversion. Joule (2025). 10.1016/j.joule.2025.102224

[4] X. Shi, K. Xu, Y. He, Z. Peng, X. Meng et al., Strategies for enhancing energy-level matching in perovskite solar cells: an energy flow perspective. Nano-Micro Lett. **17**(1), 313 (2025). 10.1007/s40820-025-01815-z 10.1007/s40820-025-01815-z PMC12187635 40553421

[5] H. Chen, C. Liu, J. Xu, A. Maxwell, W. Zhou et al., Improved charge extraction in inverted perovskite solar cells with dual-site-binding ligands. Science **384**(6692), 189–193 (2024). 10.1126/science.adm9474 38603485 10.1126/science.adm9474

[6] S. Liu, J. Li, W. Xiao, R. Chen, Z. Sun et al., Buried interface molecular hybrid for inverted perovskite solar cells. Nature **632**(8025), 536–542 (2024). 10.1038/s41586-024-07723-3 38925147 10.1038/s41586-024-07723-3

[7] C. Luo, Q. Zhou, K. Wang, X. Wang, J. He et al., Engineering bonding sites enables uniform and robust self-assembled monolayer for stable perovskite solar cells. Nat. Mater. **24**(8), 1265–1272 (2025). 10.1038/s41563-025-02275-x 40555860 10.1038/s41563-025-02275-x

[8] H. Liu, Y. Gao, F. Xu, X. Zhang, A. Ullah et al., Enhanced thermal and photostability of perovskite solar cells by a multifunctional Eu (III) trifluoromethanesulfonate additive. Adv. Funct. Mater. **34**(41), 2315843 (2024). 10.1002/adfm.202315843

[9] X. Qi, C. Song, W. Zhang, Y. Shi, Y. Gao et al., Bidirectional targeted therapy enables efficient, stable, and eco-friendly perovskite solar cells. Adv. Funct. Mater. **33**(19), 2214714 (2023). 10.1002/adfm.202214714

[10] Y. Zhan, X. Ren, S. Zhao, Z. Guo, Atomic-number-informed encoding descriptor to predict the stability of perovskite oxides and halides. Mater. Today Energy **53**, 102002 (2025). 10.1016/j.mtener.2025.102002

[11] Y. Zhan, X. Ren, J. Yi, S. Zhao, Z. Guo, A machine learning approach to identifying perovskite materials in A_2_BB′X_6_ compounds. J. Mater. Chem. C **13**(44), 22354–22364 (2025). 10.1039/d5tc03018h

[12] A. Mei, X. Li, L. Liu, Z. Ku, T. Liu et al., A hole-conductor-free, fully printable mesoscopic perovskite solar cell with high stability. Science **345**(6194), 295–298 (2014). 10.1126/science.1254763 25035487 10.1126/science.1254763

[13] Y. Fu, W. Shao, Z. Deng, W. Wu, Enabling high-efficiency ambient-air printable carbon-based large-area perovskite solar cells via effective anionic passivation. Chem. Eng. J. **503**, 158282 (2025). 10.1016/j.cej.2024.158282

[14] Y. Cheng, Z. Zheng, S. Liu, J. Xiang, C. Han et al., Scalable in-plane directional crystallization for the printable hole-conductor-free perovskite solar cell based on the carbon electrode. Adv. Energy Mater. **14**(8), 2303988 (2024). 10.1002/aenm.202303988

[15] J. Xiang, Y. Cheng, G. Zhang, Z. Liu, C. Han et al., Efficient carbon-based hole-conductor-free printable mesoscopic perovskite solar cells via a multifunctional fluorinated molecule. Adv. Funct. Mater. **34**(38), 2402816 (2024). 10.1002/adfm.202402816

[16] J. Wang, D. Wang, Y. Zhang, Y. Chen, T. Huang et al., Manipulation of Cs_0.1_FA_0.9_PbI_3_ crystallization behavior towards efficient carbon-based printable mesoscopic perovskite solar cells. J. Mater. Chem. A **12**(28), 17203–17212 (2024). 10.1039/D4TA01254B

[17] J. Xiang, C. Han, Y. Cheng, Q. Gao, W. Hu et al., Recent progress and advances of perovskite crystallization in carbon-based printable mesoscopic solar cells. Adv. Mater. **37**(9), e2415405 (2025). 10.1002/adma.202415405 39815323 10.1002/adma.202415405

[18] J. Yang, S. Li, X. Xiao, J. Du, M. Xia et al., A multifunctional piperidine-based modulator for printable mesoscopic perovskite solar cells. Chem. Eng. J. **446**, 136967 (2022). 10.1016/j.cej.2022.136967

[19] J. Xiang, C. Han, J. Qi, Y. Cheng, K. Chen et al., A polymer defect passivator for efficient hole-conductor-free printable mesoscopic perovskite solar cells. Adv. Funct. Mater. **33**(25), 2300473 (2023). 10.1002/adfm.202300473

[20] Y. Hu, S. Si, A. Mei, Y. Rong, H. Liu et al., Stable large-area (10 × 10 cm^2^) printable mesoscopic perovskite module exceeding 10% efficiency. Sol. RRL **1**(2), 1600019 (2017). 10.1002/solr.201600019

[21] J. Euvrard, Y. Yan, D.B. Mitzi, Electrical doping in halide perovskites. Nat. Rev. Mater. **6**(6), 531–549 (2021). 10.1038/s41578-021-00286-z

[22] J. Qi, J. Liu, Y. Ma, Y. Cheng, K. Chen et al., Modulating dual functionalities of hydrazide derivatives for iodide oxidation suppression and defect passivation in printable mesoscopic perovskite solar cells. Adv. Energy Mater. **14**(47), 2402344 (2024). 10.1002/aenm.202402344

[23] C. Han, J. Du, Z. Liu, Q. Gao, X. Chen et al., In situ reconstruction post-treatment for efficient carbon-based hole-conductor-free printable mesoscopic perovskite solar cells. Adv. Funct. Mater. **34**(48), 2408686 (2024). 10.1002/adfm.202408686

[24] Q. Wang, Y. Shao, H. Xie, L. Lyu, X. Liu et al., Qualifying composition dependent *p* and *n* self-doping in CH_3_NH_3_PbI_3_. Appl. Phys. Lett. **105**(16), 163508 (2014). 10.1063/1.4899051

[25] Y. Yang, X. Zou, Y. Pei, X. Bai, W. Jin et al., Effect of doping of NaI monovalent cation halide on the structural, morphological, optical and optoelectronic properties of MAPbI_3_ perovskite. J. Mater. Sci. Mater. Electron. **29**(1), 205–210 (2018). 10.1007/s10854-017-7905-3

[26] Y. Chen, H. Jing, F. Ling, W. Kang, T. Zhou et al., Tuning the electronic structures of all-inorganic lead halide perovskite CsPbI_3_ via heterovalent doping: a first-principles investigation. Chem. Phys. Lett. **722**, 90–95 (2019). 10.1016/j.cplett.2019.02.050

[27] T. Shi, W.-J. Yin, Y. Yan, Predictions for p-type CH_3_NH_3_PbI_3_ perovskites. J. Phys. Chem. C **118**(44), 25350–25354 (2014). 10.1021/jp508328u

[28] E. Mosconi, B. Merabet, D. Meggiolaro, A. Zaoui, F. De Angelis, First-principles modeling of bismuth doping in the MAPbI_3_ perovskite. J. Phys. Chem. C **122**(25), 14107–14112 (2018). 10.1021/acs.jpcc.8b01307

[29] M. Abdi-Jalebi, M.I. Dar, A. Sadhanala, S.P. Senanayak, M. Franckevičius et al., Impact of monovalent cation halide additives on the structural and optoelectronic properties of CH_3_NH_3_PbI_3_ perovskite. Adv. Energy Mater. **6**(10), 1502472 (2016). 10.1002/aenm.201502472

[30] A.L. Abdelhady, M.I. Saidaminov, B. Murali, V. Adinolfi, O. Voznyy et al., Heterovalent dopant incorporation for bandgap and type engineering of perovskite crystals. J. Phys. Chem. Lett. **7**(2), 295–301 (2016). 10.1021/acs.jpclett.5b02681 26727130 10.1021/acs.jpclett.5b02681

[31] Y. Yamada, M. Hoyano, R. Akashi, K. Oto, Y. Kanemitsu, Impact of chemical doping on optical responses in bismuth-doped CH_3_NH_3_PbBr_3_ single crystals: carrier lifetime and photon recycling. J. Phys. Chem. Lett. **8**(23), 5798–5803 (2017). 10.1021/acs.jpclett.7b02508 29130309 10.1021/acs.jpclett.7b02508

[32] R. Meng, G. Wu, J. Zhou, H. Zhou, H. Fang et al., Understanding the impact of bismuth heterovalent doping on the structural and photophysical properties of CH_3_NH_3_PbBr_3_ halide perovskite crystals with near-IR photoluminescence. Chem. Eur. J. **25**(21), 5480–5488 (2019). 10.1002/chem.201805370 30770600 10.1002/chem.201805370

[33] A.M. Ulatowski, A.D. Wright, B. Wenger, L.R.V. Buizza, S.G. Motti et al., Charge-carrier trapping dynamics in bismuth-doped thin films of MAPbBr_3_ perovskite. J. Phys. Chem. Lett. **11**(9), 3681–3688 (2020). 10.1021/acs.jpclett.0c01048 32302145 10.1021/acs.jpclett.0c01048

[34] E.M. Miller, Y. Zhao, C.C. Mercado, S.K. Saha, J.M. Luther et al., Substrate-controlled band positions in CH_3_NH_3_PbI_3_ perovskite films. Phys. Chem. Chem. Phys. **16**(40), 22122–22130 (2014). 10.1039/c4cp03533j 25209217 10.1039/c4cp03533j

[35] S. Olthof, K. Meerholz, Substrate-dependent electronic structure and film formation of MAPbI_3_ perovskites. Sci. Rep. **7**, 40267 (2017). 10.1038/srep40267 28084313 10.1038/srep40267 PMC5234022

[36] Y. Meng, Z. Lai, F. Li, W. Wang, S. Yip et al., Perovskite core-shell nanowire transistors: interfacial transfer doping and surface passivation. ACS Nano **14**(10), 12749–12760 (2020). 10.1021/acsnano.0c03101 32910641 10.1021/acsnano.0c03101

[37] N.K. Noel, S.N. Habisreutinger, B. Wenger, Y.-H. Lin, F. Zhang et al., Elucidating the role of a tetrafluoroborate-based ionic liquid at the n-type oxide/perovskite interface. Adv. Energy Mater. **10**(4), 1903231 (2020). 10.1002/aenm.201903231

[38] W. Xiong, W. Tang, G. Zhang, Y. Yang, Y. Fan et al., Controllable p- and n-type behaviours in emissive perovskite semiconductors. Nature **633**(8029), 344–350 (2024). 10.1038/s41586-024-07792-4 39261614 10.1038/s41586-024-07792-4

[39] T. Du, S. Qiu, X. Zhou, V.M. Le Corre, M. Wu et al., Efficient, stable, and fully printed carbon-electrode perovskite solar cells enabled by hole-transporting bilayers. Joule **7**(8), 1920–1937 (2023). 10.1016/j.joule.2023.06.005

[40] J. Liu, X. Chen, K. Chen, W. Tian, Y. Sheng et al., Electron injection and defect passivation for high-efficiency mesoporous perovskite solar cells. Science **383**(6688), 1198–1204 (2024). 10.1126/science.adk9089 38484055 10.1126/science.adk9089

[41] M. Burgelman, P. Nollet, S. Degrave, Modelling polycrystalline semiconductor solar cells. Thin Solid Films **361**, 527–532 (2000). 10.1016/S0040-6090(99)00825-1

[42] S. Karthick, S. Velumani, J. Bouclé, Experimental and SCAPS simulated formamidinium perovskite solar cells: a comparison of device performance. Sol. Energy **205**, 349–357 (2020). 10.1016/j.solener.2020.05.041

[43] Z. Ni, C. Bao, Y. Liu, Q. Jiang, W.-Q. Wu et al., Resolving spatial and energetic distributions of trap states in metal halide perovskite solar cells. Science **367**(6484), 1352–1358 (2020). 10.1126/science.aba0893 32193323 10.1126/science.aba0893

[44] S. Taheri, A. Ahmadkhan kordbacheh, M. Minbashi, A. Hajjiah, Effect of defects on high efficient perovskite solar cells. Opt. Mater. **111**, 110601 (2021). 10.1016/j.optmat.2020.110601

[45] Y. Zhang, Z. Yang, T. Ma, Z. Ai, C. Wang et al., A theoretical investigation of transport layer-free homojunction perovskite solar cells *via* a detailed photoelectric simulation. Adv. Energy Mater. **13**(12), 2203366 (2023). 10.1002/aenm.202203366

[46] J. Jin, J. Zhang, J. Zhang, S. Zou, Y. Xin et al., Semi-transparent perovskite solar cells with high light-utilisation efficiency of 5.10% fabricated through molecular dipole engineering. Adv. Energy Mater. **15**(38), e01994 (2025). 10.1002/aenm.202501994

[47] H. Lv, J. Liu, P. Wang, G. Hou, Y. Zhao et al., A dual-functional coordination additive for defect passivation and large-grain formation in perovskite film. Adv. Funct. Mater. **35**(51), e10831 (2025). 10.1002/adfm.202510831

[48] I. Mora-Seró, How do perovskite solar cells work? Joule **2**(4), 585–587 (2018). 10.1016/j.joule.2018.03.020

[49] F.H. Isikgor, S. Zhumagali, L.V.T. Merino, M. De Bastiani, I. McCulloch et al., Molecular engineering of contact interfaces for high-performance perovskite solar cells. Nat. Rev. Mater. **8**(2), 89–108 (2023). 10.1038/s41578-022-00503-3

[50] C. Wang, J. Xiang, J. Liu, C. Han, Z. Zheng et al., Bifunctional compound induced dual back surface fields for efficient hole transport layer-free perovskite solar cells. Adv. Mater. **37**(27), 2502724 (2025). 10.1002/adma.202502724 10.1002/adma.202502724 40331473

[51] Z. Ni, S. Xu, H. Jiao, H. Gu, C. Fei et al., High grain boundary recombination velocity in polycrystalline metal halide perovskites. Sci. Adv. **8**(36), eabq8345 (2022). 10.1126/sciadv.abq8345 36070394 10.1126/sciadv.abq8345 PMC9451161

[52] Q. Sun, Z. Yin, S. Wang, C. Zhao, J. Leng et al., Long-range exciton transport in perovskite–metal organic framework solid composites. J. Phys. Chem. Lett. **11**(21), 9045–9050 (2020). 10.1021/acs.jpclett.0c02974 33044078 10.1021/acs.jpclett.0c02974

[53] Y. Yin, W. Tian, J. Leng, J. Bian, S. Jin, Carrier transport limited by trap state in Cs_2_AgBiBr_6_ double perovskites. J. Phys. Chem. Lett. **11**(17), 6956–6963 (2020). 10.1021/acs.jpclett.0c01817 32787195 10.1021/acs.jpclett.0c01817

[54] Z. Shi, Z. Ni, J. Huang, Direct observation of fast carriers transport along out-of-plane direction in a Dion–jacobson layered perovskite. ACS Energy Lett. **7**(3), 984–987 (2022). 10.1021/acsenergylett.2c00098

[55] C. He, Z. Zheng, H. Tang, L. Zhao, F. Lu, Electrochemical impedance spectroscopy characterization of electron transport and recombination in ZnO nanorod dye-sensitized solar cells. J. Phys. Chem. C **113**(24), 10322–10325 (2009). 10.1021/jp902523c

[56] R. Tian, C. Liu, Y. Meng, Y. Wang, R. Cao et al., Nucleation regulation and mesoscopic dielectric screening in α-FAPbI_3_. Adv. Mater. **36**(13), e2309998 (2024). 10.1002/adma.202309998 38108580 10.1002/adma.202309998

[57] Y. Wen, T. Zhang, X. Wang, T. Liu, Y. Wang et al., Amorphous (lysine)_2_PbI_2_ layer enhanced perovskite photovoltaics. Nat. Commun. **15**(1), 7085 (2024). 10.1038/s41467-024-51551-y 39154032 10.1038/s41467-024-51551-y PMC11330473

[58] Y. Huang, K. Yan, X. Wang, B. Li, B. Niu et al., High-efficiency inverted perovskite solar cells via in situ passivation directed crystallization. Adv. Mater. **36**(41), e2408101 (2024). 10.1002/adma.202408101 39140642 10.1002/adma.202408101

[59] J. Shi, Y. Li, Y. Li, D. Li, Y. Luo et al., From ultrafast to ultraslow: charge-carrier dynamics of perovskite solar cells. Joule **2**(5), 879–901 (2018). 10.1016/j.joule.2018.04.010

[60] R. Tang, Z.-H. Zheng, Z.-H. Su, X.-J. Li, Y.-D. Wei et al., Highly efficient and stable planar heterojunction solar cell based on sputtered and post-selenized Sb_2_Se_3_ thin film. Nano Energy **64**, 103929 (2019). 10.1016/j.nanoen.2019.103929

[61] Y. Gao, Z. Xiao, M. Cui, M.I. Saidaminov, F. Tan et al., Asymmetric Π-bridge engineering enables high-permittivity benzo [1, 2-B: 4, 5-b′] difuran-conjugated polymer for efficient organic solar cells. Adv. Mater. **36**(9), 2306373 (2024). 10.1002/adma.202306373 10.1002/adma.202306373 37703387

[62] S. Xiong, F. Tian, F. Wang, A. Cao, Z. Chen et al., Reducing nonradiative recombination for highly efficient inverted perovskite solar cells *via* a synergistic bimolecular interface. Nat. Commun. **15**(1), 5607 (2024). 10.1038/s41467-024-50019-3 38965277 10.1038/s41467-024-50019-3 PMC11224317

[63] A. Guerrero, A. Bou, G. Matt, O. Almora, T. Heumüller et al., Switching off hysteresis in perovskite solar cells by fine-tuning energy levels of extraction layers. Adv. Energy Mater. **8**(21), 1703376 (2018). 10.1002/aenm.201703376

[64] D.-H. Kang, N.-G. Park, On the current-voltage hysteresis in perovskite solar cells: dependence on perovskite composition and methods to remove hysteresis. Adv. Mater. **31**(34), e1805214 (2019). 10.1002/adma.201805214 30773704 10.1002/adma.201805214

[65] S. Wang, H. Jiang, Z. Han, C. Wang, Y. Cao et al., Defection-stripping and balanced carrier extraction strategy enables efficient and ultra-low hysteresis of inverted inorganic perovskite solar cells. Chem. Eng. Sci. **302**, 120891 (2025). 10.1016/j.ces.2024.120891

