# Supplementary material for: Novel Gradient p-Doping Strategy Enables Efficient Carbon-Based Hole Transport Layer-Free Perovskite Solar Cells
Source: Nanomicro Lett. 2026 Feb 23;18:258. doi: 10.1007/s40820-026-02112-z (PMC12929742; doi:10.1007/s40820-026-02112-z)
Supplement: Supplementary file 1 — Supplementary file1 (DOCX 19931 KB) [file 40820_2026_2112_MOESM1_ESM.docx]

Supporting Information for

**Novel Gradient p-Doping Strategy Enables Efficient Carbon-Based Hole Transport Layer-Free Perovskite Solar Cells**

Junwei Xiang^1,^ †, Siqi Jiang^1,^ †, Yanjie Cheng^1^, Weiting Du^1^, Yuan Shi^1^, Song Shen^1^, Bolun Zhang^1^, Qian Yue^1^, Xinyi Xu^1^, Anyi Mei^1^, Yang Zhou^1, *^, Yinhua Zhou^1^, Hongwei Han^1, *^

*^1^* Michael Grätzel Center for Mesoscopic Solar Cells, Wuhan National Laboratory for Optoelectronics, Key Laboratory of Materials Chemistry for Energy Conversion and Storage of Ministry of Education, Huazhong University of Science and Technology, Wuhan, Hubei 430074, P. R. China

**†**Junwei Xiang and Siqi Jiang contributed equally to this work.

***** Corresponding authors. E-mails: [yangz@hust.edu.cn](mailto:yangz@hust.edu.cn) (Yang Zhou); [hongwei.han@mail.hust.edu.cn](mailto:hongwei.han@mail.hust.edu.cn) (Hongwei Han)

**S1 Characterization**

Scanning electron microscopy (SEM) images were obtained using a Nova NanoSEM 450 field-emission SEM. X-ray diffraction (XRD) patterns were recorded on an X’Pert PRO diffractometer using Cu Kα radiation (λ = 1.5406Å) at 40kV and 40mA, scanned from 10° to 60° at a rate of 10° min⁻¹. X-ray photoelectron spectroscopy (XPS) was performed using a Kratos Axis-Ultra DLD-600 W system. UV-visible absorption spectra were measured using a SolidSpec-3700 UV–vis–NIR spectrophotometer. The simulated AM 1.5G solar spectrum with an intensity of 100 mW cm^-2^ was calibrated using a certified monocrystalline silicon reference cell (Newport, model 91150V). *J*-*V* characteristics were measured from 1.2V to -0.2V at a scan rate of 100mV s^-1^, using a mask with a circular aperture of 0.1018cm^2^. Incident photon-to-current conversion efficiency (IPCE) was recorded using a 150W xenon lamp (Oriel) with a Cornerstone 74004 monochromator. Electrochemical impedance spectroscopy (EIS) and thermal admittance spectroscopy (TAS) was carried out under dark and open-circuit conditions using a ZAHNER Zennium electrochemical workstation over a frequency range of 100mHz to 1MHz. Transient photocurrent (TPC) measurements were carried out using a CHI1000C multichannel electrochemical workstation. The TOF-SIMS depth profiling was conducted using a GAIA3 TOF-SIMS 5-100 spectrometer (ION-TOF GmbH). The work function was measured using a Kelvin probe scanning system (SKP5050, Kerui Company). PL intensity and lifetime mappings were conducted using a multifunctional laser scanning confocal fluorescence imaging system (FLIM300, TIME-TECH SPECTRA). A 405nm pulsed laser with a repetition rate of 5MHz and an excitation intensity of 100mW⋅cm^-2^ was used.

**S2 Sample Preparation**

For XRD samples, the carbon layer of p-MPSCs was mechanically removed using tape prior to test. For TOF-SIMS sample, p-MPSCs were sent as prepared. For the PL mapping samples, to prevent the influence of moisture and oxygen during measurements, the device cross-section was well encapsulated by cover glass and UV-curable resin. In the case of XPS and UV-vis samples, the perovskite thin films were prepared by the two-step spin coating method. The perovskite precursor solution was deposited on the FTO glass at 1,000 rpm for 10 seconds and 6,000 rpm for 30 seconds with accelerations of 200 and 2,000 rpm, respectively. In the second step, 70μL of chlorobenzene anti-solvent was dropped onto the spinning substrate. The resulting sample was dried on a hot plate at 100 °C for 30 minutes before the measurement.

**S3 Device Stability Measurement**

The device was encapsulated by hot-pressing a cover glass and EVA gel to ensure a robust seal. Following encapsulation, it was subjected to accelerated aging in a solar simulation chamber under controlled environmental conditions: an ambient temperature of (55 ± 5) °C, relative humidity of (55 ± 5) %, and continuous illumination from a halogen lamp at 100 mW⋅cm^-2^ without a UV filter. During the aging process, the device’s performance was continuously monitored at its maximum power point to assess its long-term operational stability.

**S4 Supplementary Figures and Tables**


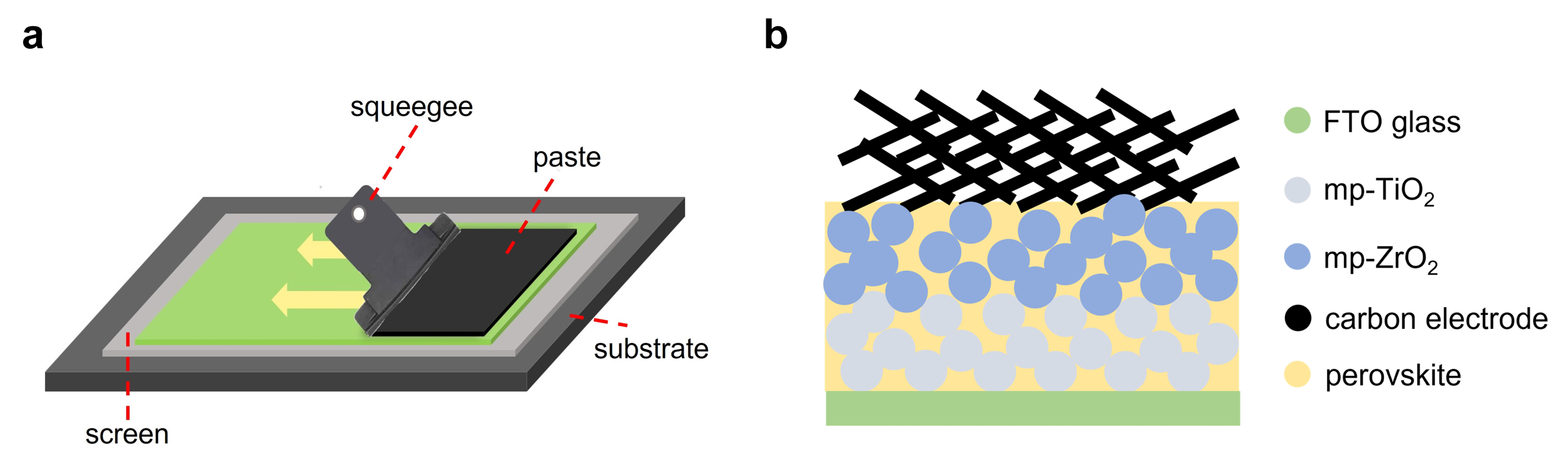


**Fig. S1** **a** Schematic of screen-printing technique used for fabricating mp-TiO_2_/mp-ZrO_2_/porous carbon scaffold. **b** Schematic of the p-MPSC structure

**Fig. S2** Molecular structure of PCPA


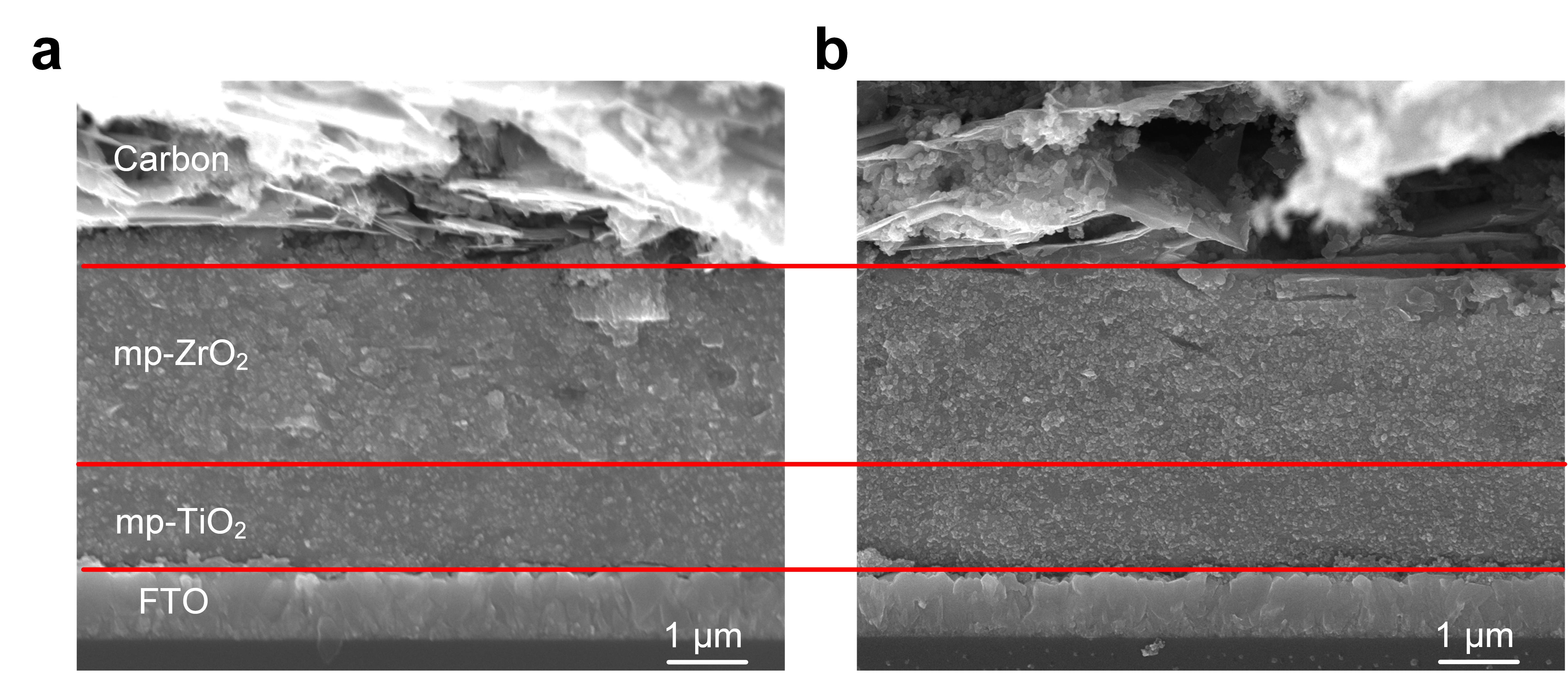


**Fig. S3** Cross-sectional SEM images of p-MPSCs **a** without and **b** with PCPA


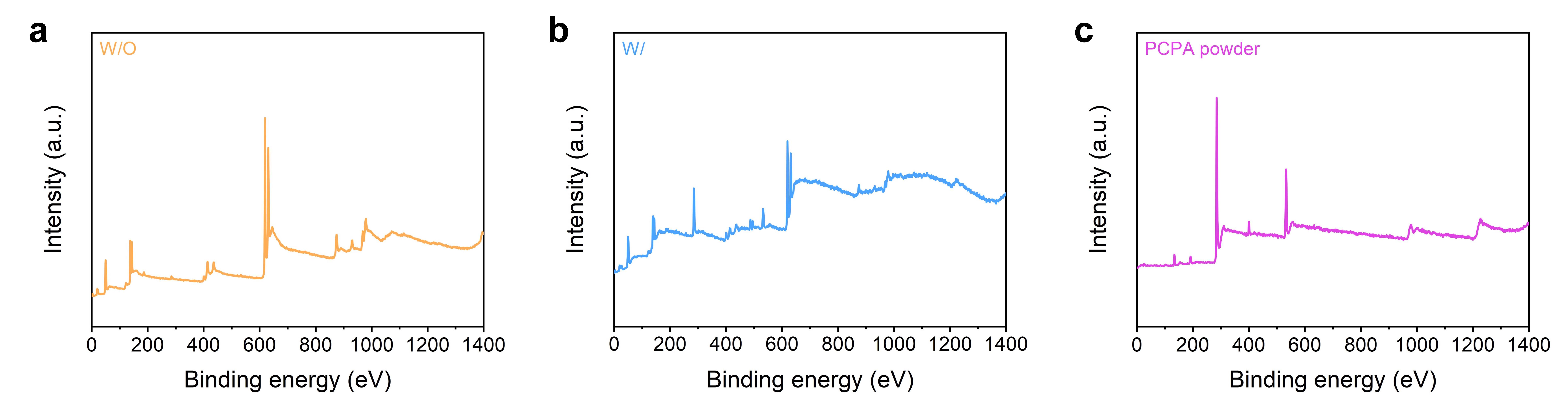


**Fig. S4** Full XPS spectra of perovskite films **a** without and **b** with PCPA, and **c** pure PCPA powder


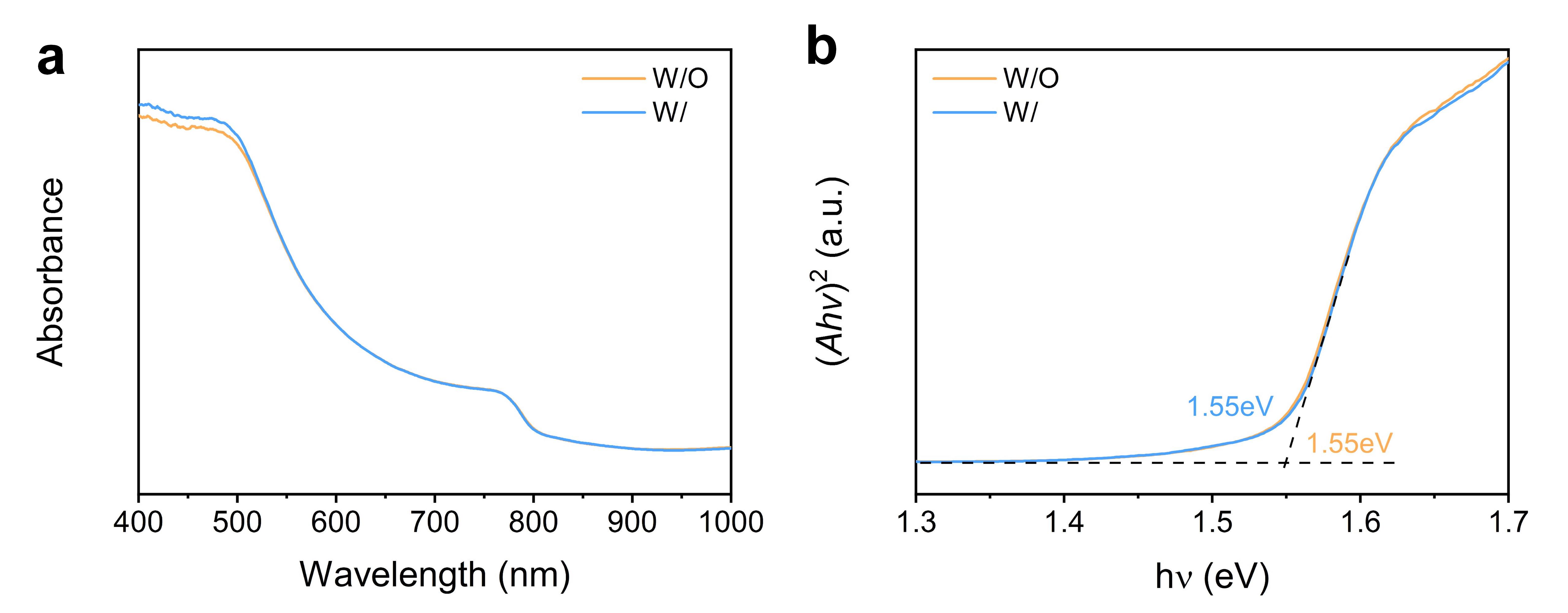


**Fig. S5** **a** UV-vis spectra and **b** Tauc plots of perovskite thin films without and with PCPA


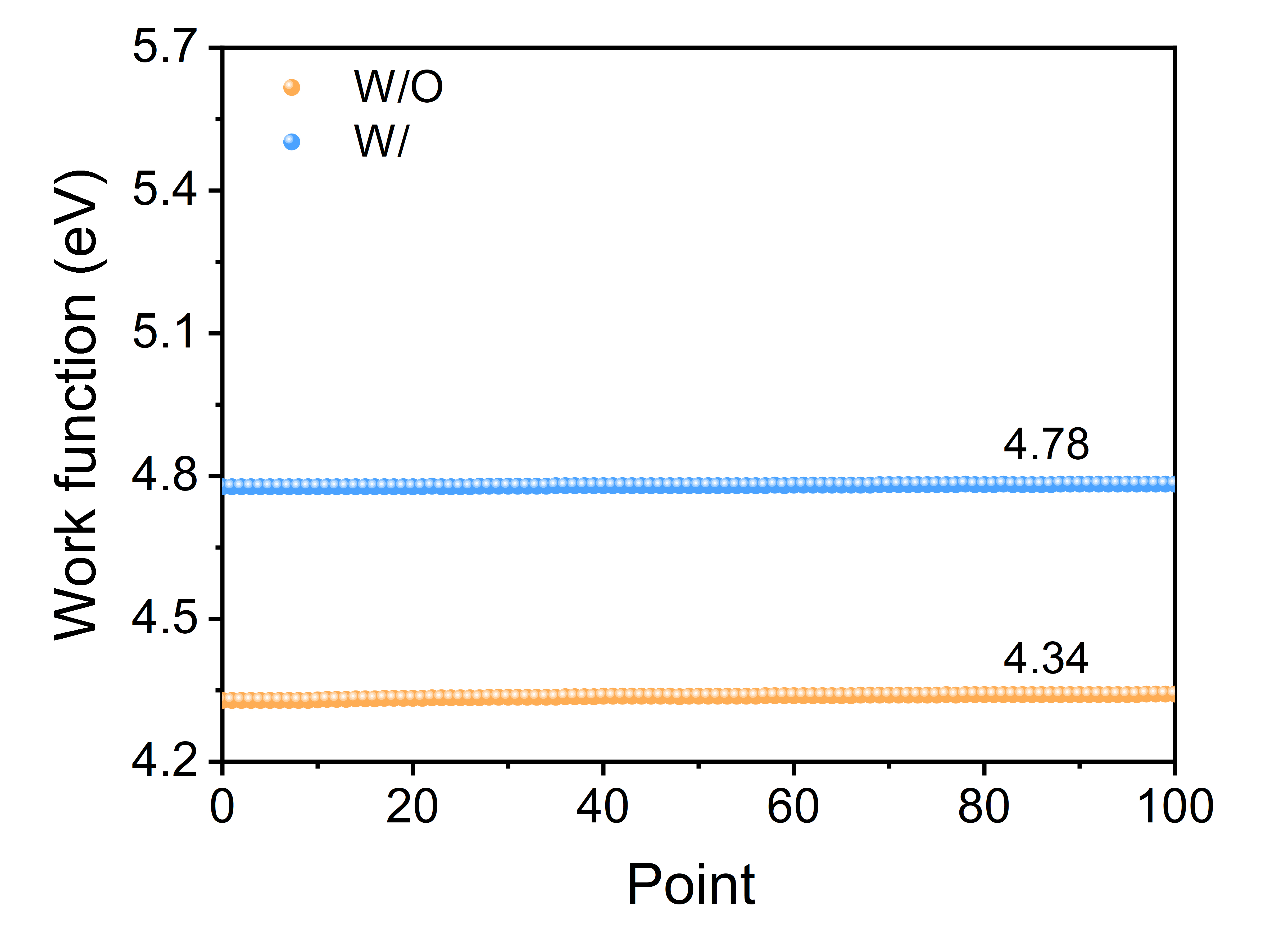


**Fig. S6** *W*_F_ of perovskite film without and with PCPA obtained by Kelvin Probe test

**Table S1** Detailed parameters of each layer in the PSC used for simulations [S1-S4]

| Material parameter | TiO_2_ | Perovskite-1 | Perovskite-2 |
| --- | --- | --- | --- |
| Thickness (nm) | 50 | 800 | 2000 |
| Bandgap *E*_g_ (eV) | 3.20 | 1.55 | 1.55 |
| Electron affinity *χ* (eV) | 4.10 | 3.88 | 3.88 |
| Relative dielectric constant *ε*_r_ | 100 | 30 | 30 |
| Effective CB density *N*_C_ (cm^-3^) | 1×10^21^ | 2.0×10^18^ | 2.0×10^18^ |
| Effective VB density *N*_V_ (cm^-3^) | 2×10^20^ | 1.8×10^19^ | 1.8×10^19^ |
| Electron mobility *µ*_n_ (cm^2^/V/s) | 6×10^-3^ | 50 | 5 |
| Hole mobility *µ*_p_ (cm^2^/V/s) | 6×10^-3^ | 50 | 50 |
| Acceptor doping concentration *N*_A_ (cm^-3^) | 0 | 0 | 0-1×10^17^（linearly increasing） |
| Donor doping concentration *N*_D_ (cm^-3^) | 5×10^20^ | 1×10^14^ | 1×10^14^ |
| Radiation recombination coefficient *r* (cm^-3^/s) | 0 | 1×10^-11^ | 1×10^-11^ |
| Charged defect type | Positive | Negative; Positive | Negative; Positive |
| Electron capture cross section *σ*_-_ (cm^2^) | 1×10^-18^ | 10^-16^；10^-15^ | 10^-16^；10^-15^ |
| Hole capture cross section *σ*_+_ (cm^2^) | 1×10^-19^ | 10^-15^；10^-16^ | 10^-15^；10^-16^ |
| Distribution form of defect energy level | Gaussian | Gaussian | Gaussian |
| Defect energy level position | Above VB | Above VB； Below CB | Above VB； Below CB |
| Depth of defect energy level *E*_t_ (eV) | 0.6 | 0.15；0.45 | 0.15；0.45 |
| Defect state density *N*_t_ (cm^-3^) | 1×10^17^ | 5×10^15^；1×10^14^ | 5×10^15^；1×10^14^ |

**Table S2** Defect densities at the interfaces for simulations [S1-S4]

| Interface defect parameter | TiO_2_ / Perovskite |
| --- | --- |
| Charged defect type | Positive |
| Electron capture cross section *σ*_-_ (cm^2^) | 1×10^-18^ |
| Hole capture cross section *σ*_+_ (cm^2^) | 1×10^-19^ |
| Distribution form of defect energy level | Single |
| Defect energy level position | Below CB |
| Depth of defect energy level *E*_t_ (eV) | 0.32 |
| Defect state density *N*_t_ (cm^-3^) | 1×10^12^ |


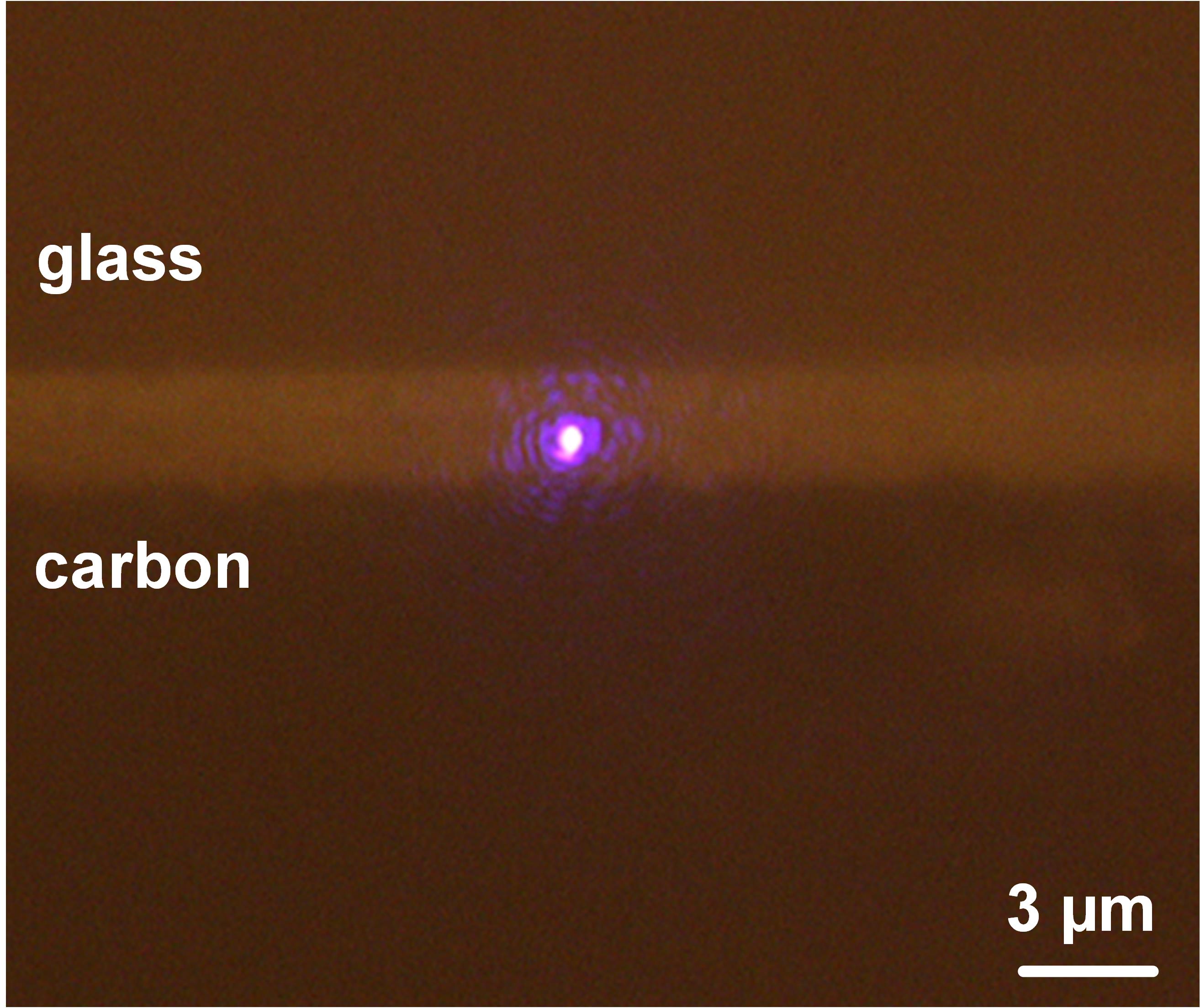


**Fig. S7** Photo image of exciting a single point in mp-ZrO_2_ region by a 405nm pulsed laser for analyzing the carrier diffusion behavior in mp-ZrO_2_





**Fig. S8** PL intensity images of the perovskite within the mp-ZrO_2_ layer at different time intervals without and with PCPA


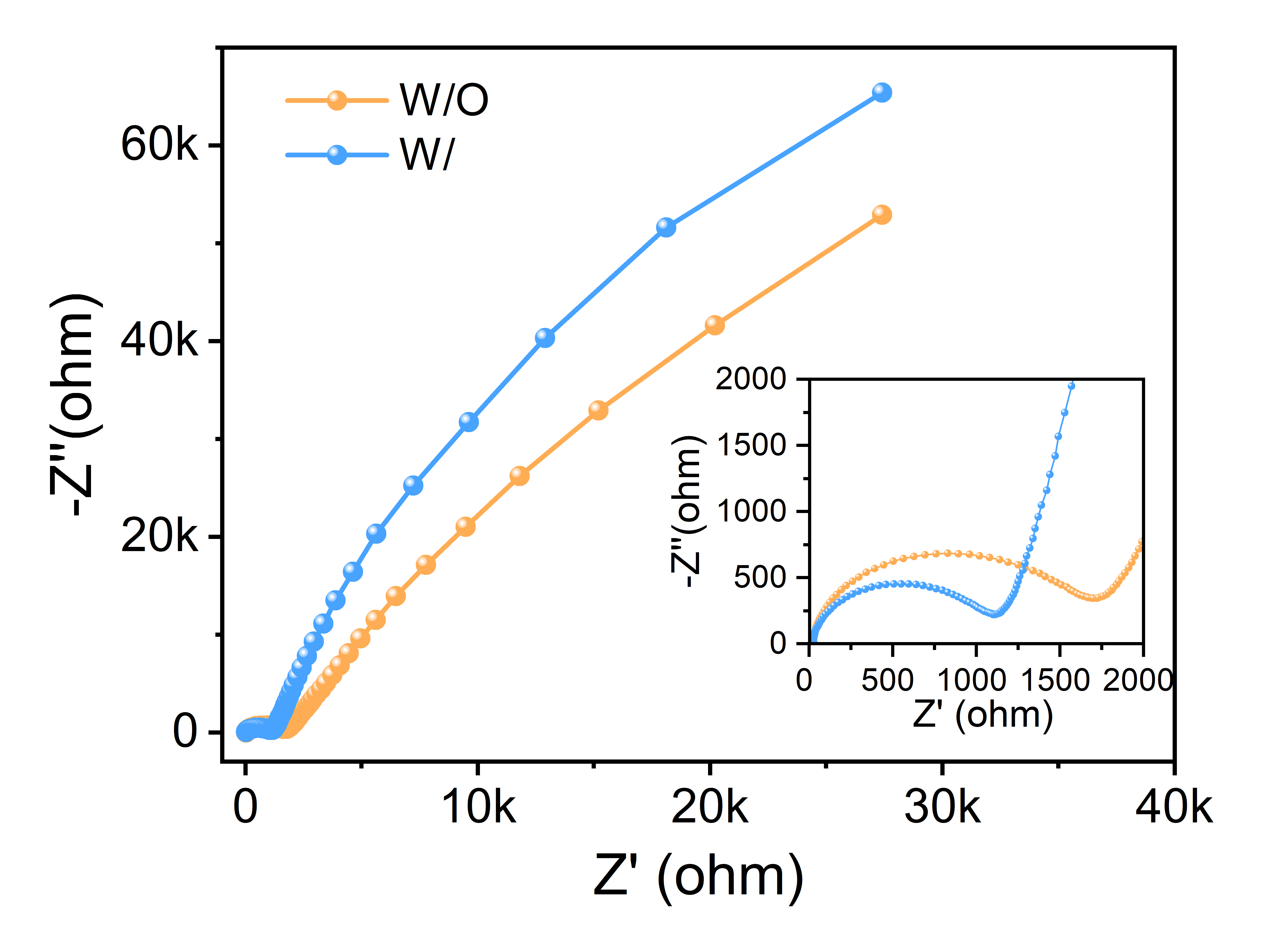


**Fig. S9** Nyquist plots of p-MPSCs without and with PCPA


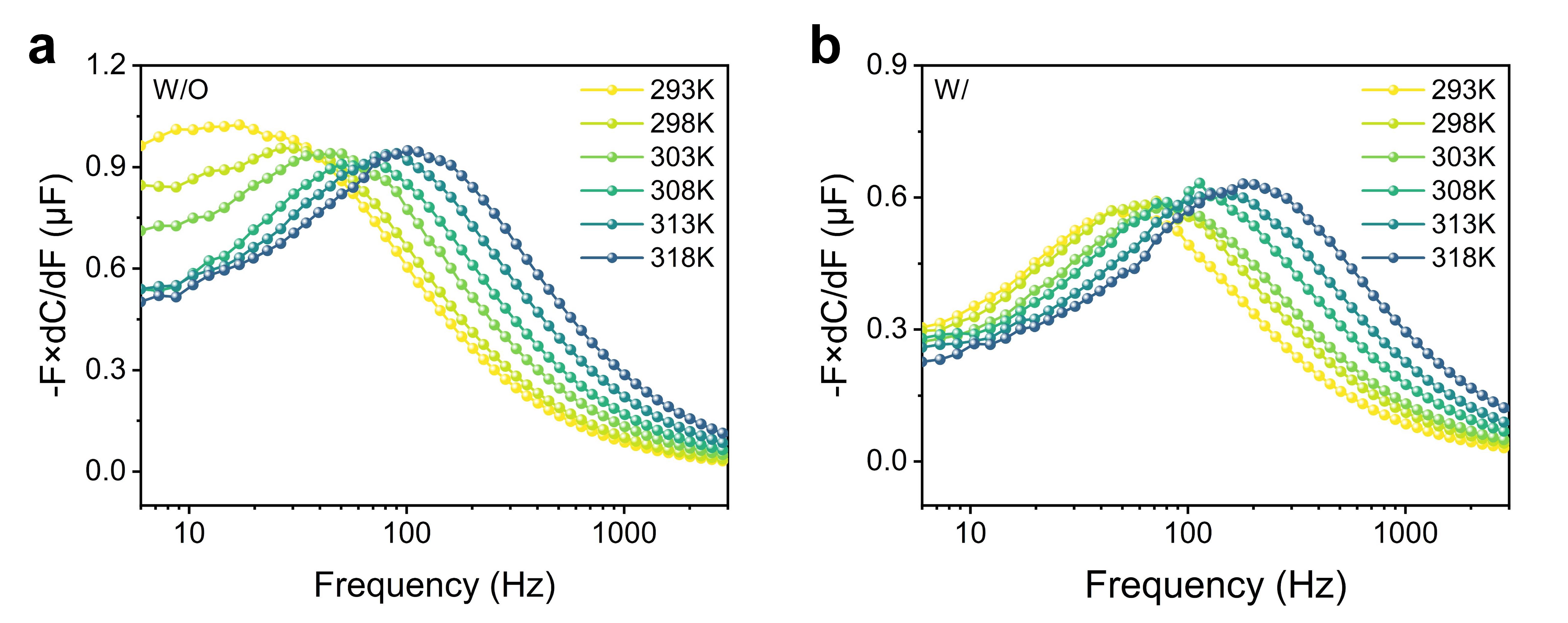


**Fig. S10** The temperature dependent -F🞨dC/dF plots derived from the temperature dependent C-F plots of devices without and with PCPA


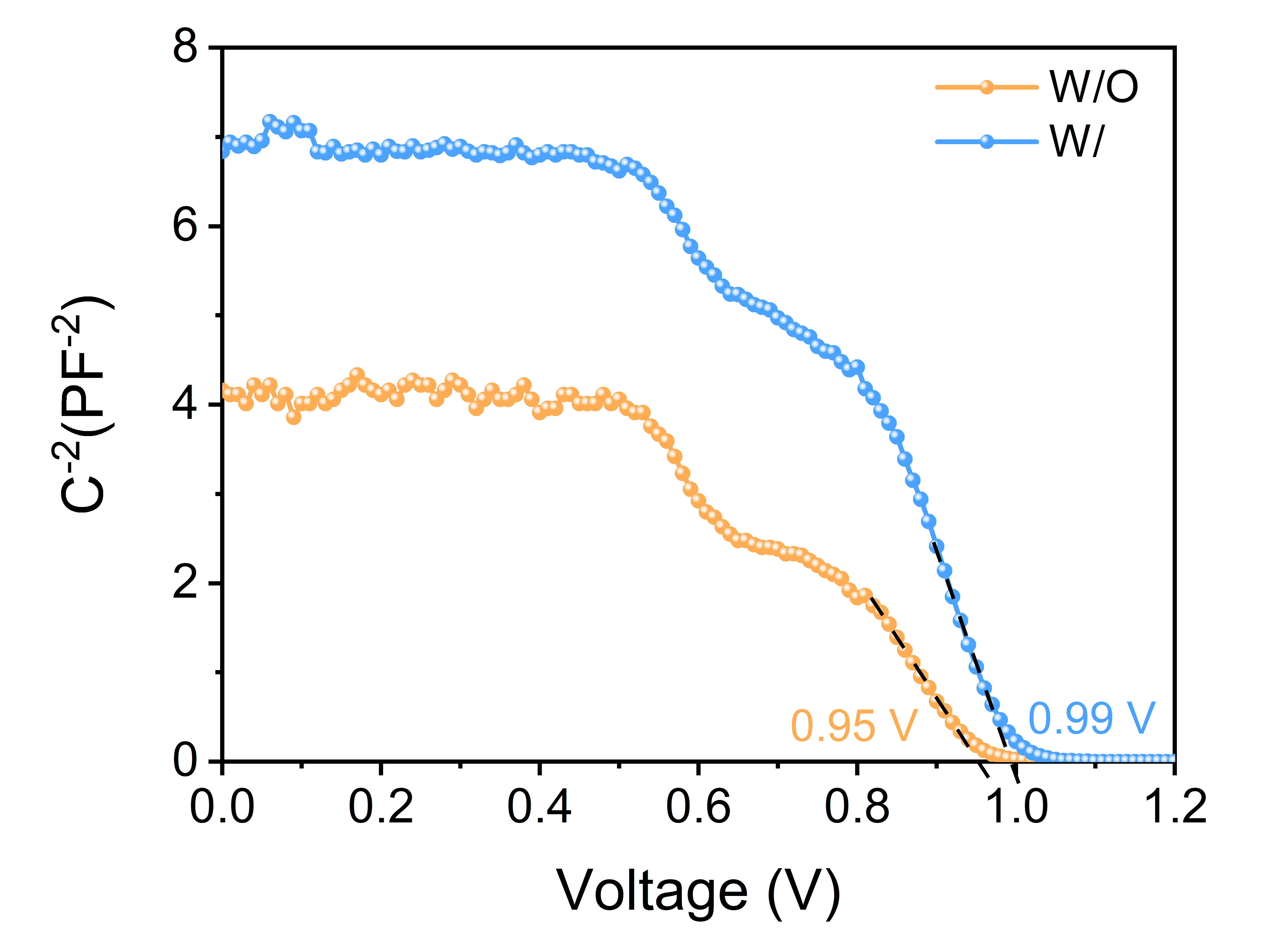


**Fig. S11** Mott-Schottky plots of p-MPSCs without and with PCPA


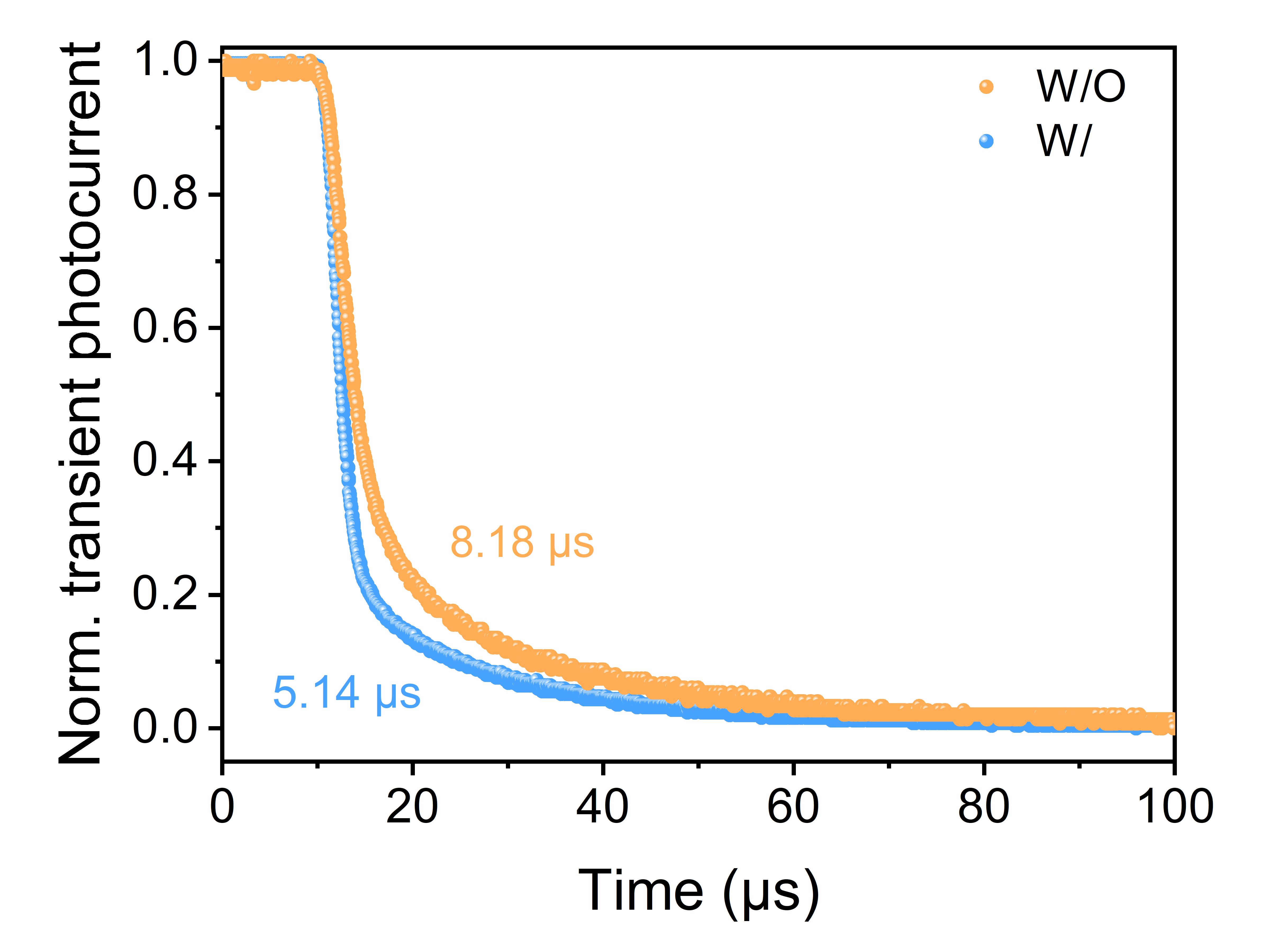


**Fig. S12** TPC decay curves of p-MPSCs without and with PCPA

**Table S3** Fitted TPC results of p-MPSCs without and with PCPA

|  | ***τ*_1_ (µs)** | ***A*_1_ (%)** | ***τ*_2_ (µs)** | ***A*_2_ (%)** | ***τ*_ave_ (µs)** |
| --- | --- | --- | --- | --- | --- |
| **control** | 3.42 | 77.00 | 24.10 | 23.00 | 8.18 |
| **with PCPA** | 2.08 | 84.05 | 21.30 | 15.95 | 5.14 |

The average carrier lifetime is calculated by the equation:

$$\tau_{ave}=\frac{A_{1}\tau_{1}+A_{2}\tau_{2}}{A_{1}+A_{2}}$$

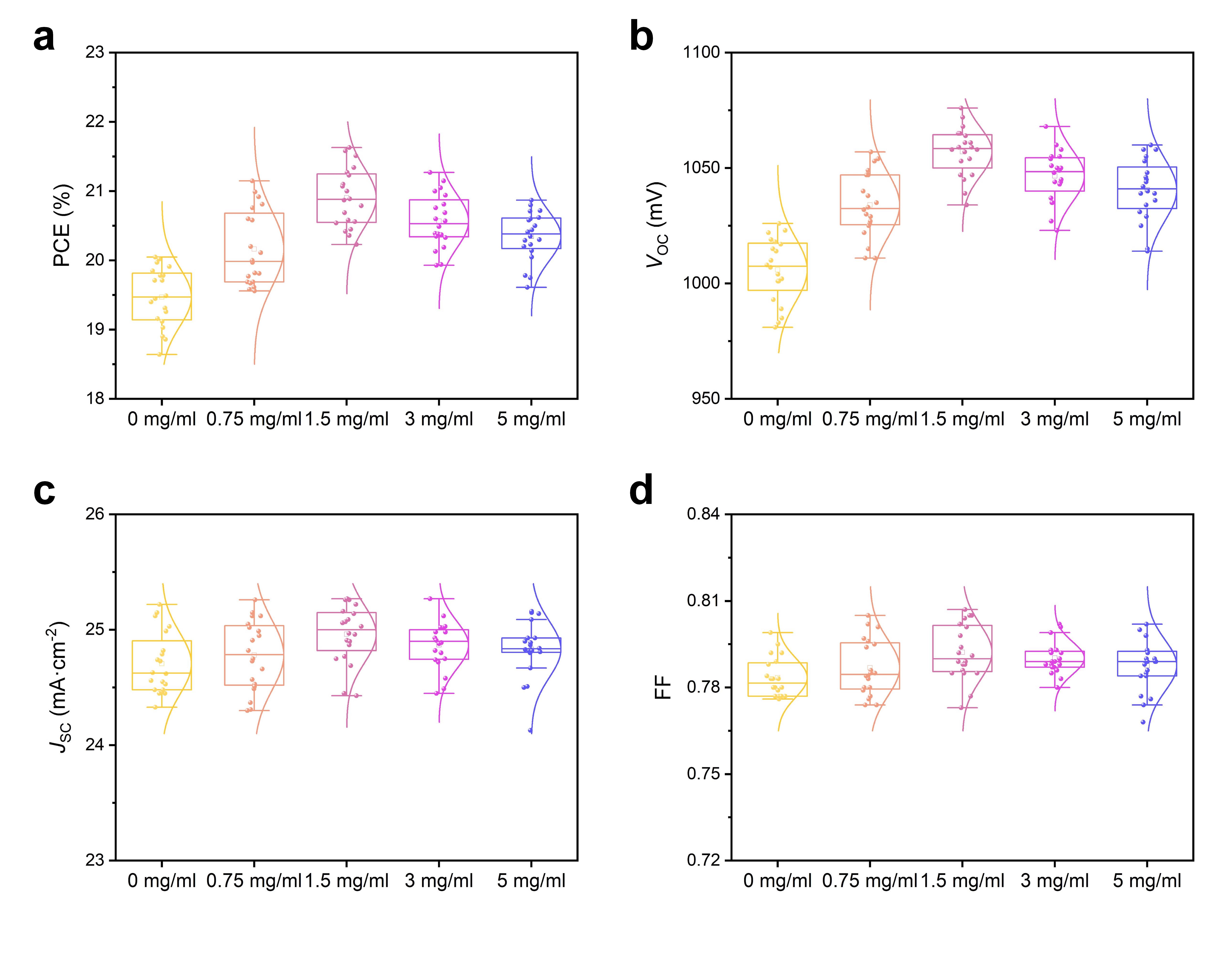


**Fig. S13** Statistics of PV parameters of p-MPSCs fabricated with varied concentrations of PCPA (20 devices per group)

**Table S4** Averages of PV parameters of devices fabricated with varied concentrations of PCPA (20 devices per group)

| **concentration** | ***V*_OC_ (mV)** | ***J*_SC_ (mA cm^-2^)** | **FF** | **PCE (%)** |
| --- | --- | --- | --- | --- |
| 0 mg/ml  0.75 mg/ml  1.5 mg/ml  3 mg/ml | 1005.90±13.54  1034.05±13.62  1057.10±10.36  1046.55±10.83 | 24.71±0.26  24.78±0.29  24.96±0.24  24.87±0.20 | 0.783±0.007  0.787±0.009  0.792±0.009  0.790±0.005 | 19.47±0.41  20.16±0.53  20.91±0.42  20.57±0.38 |
| 5 mg/ml | 1040.55±12.97 | 24.83±0.24 | 0.788±0.009 | 20.35±0.34 |


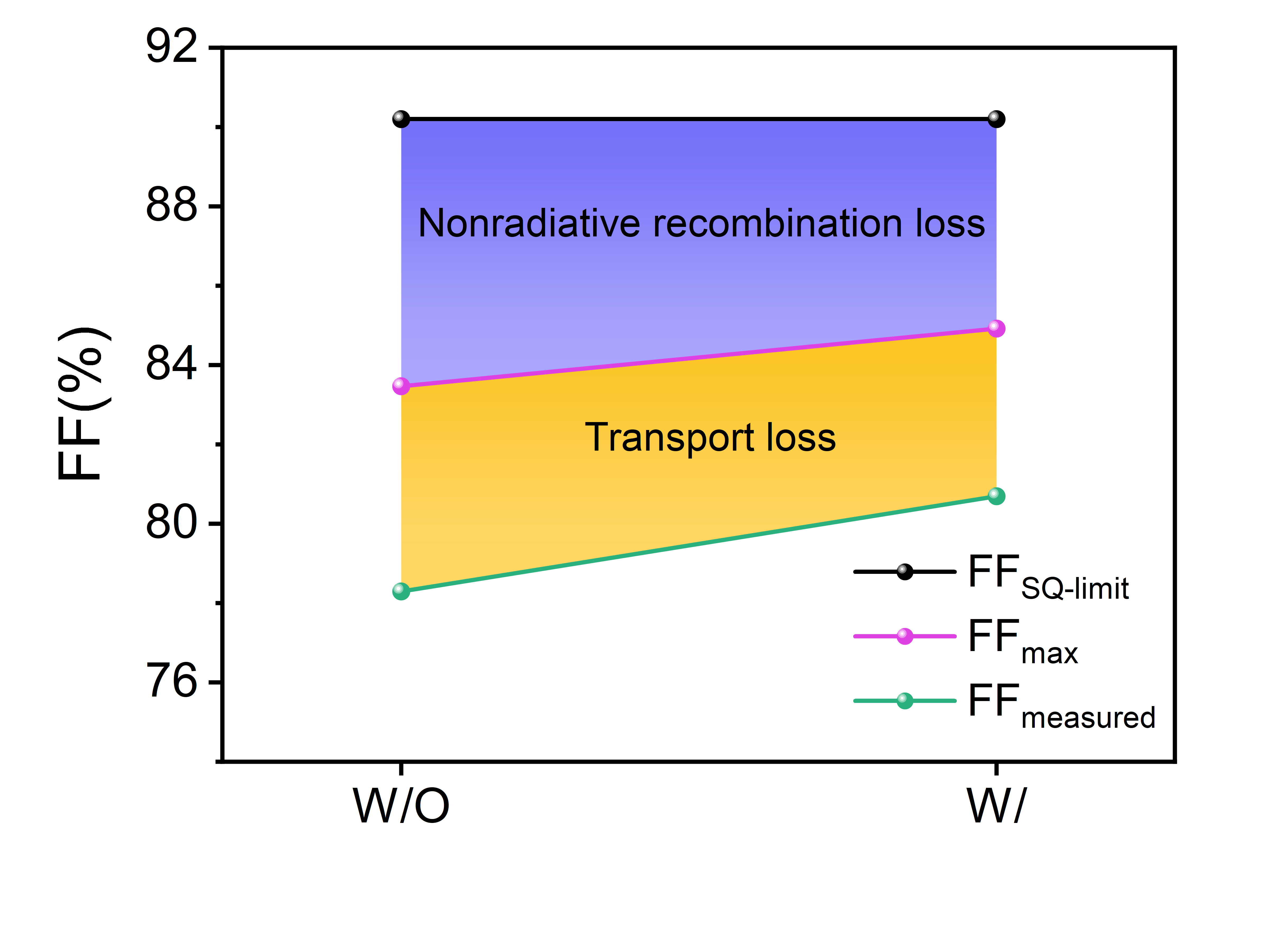


**Fig. S14** FF Analysis of nonradiative recombination loss and transport loss for p-MPSCs without and with PCPA


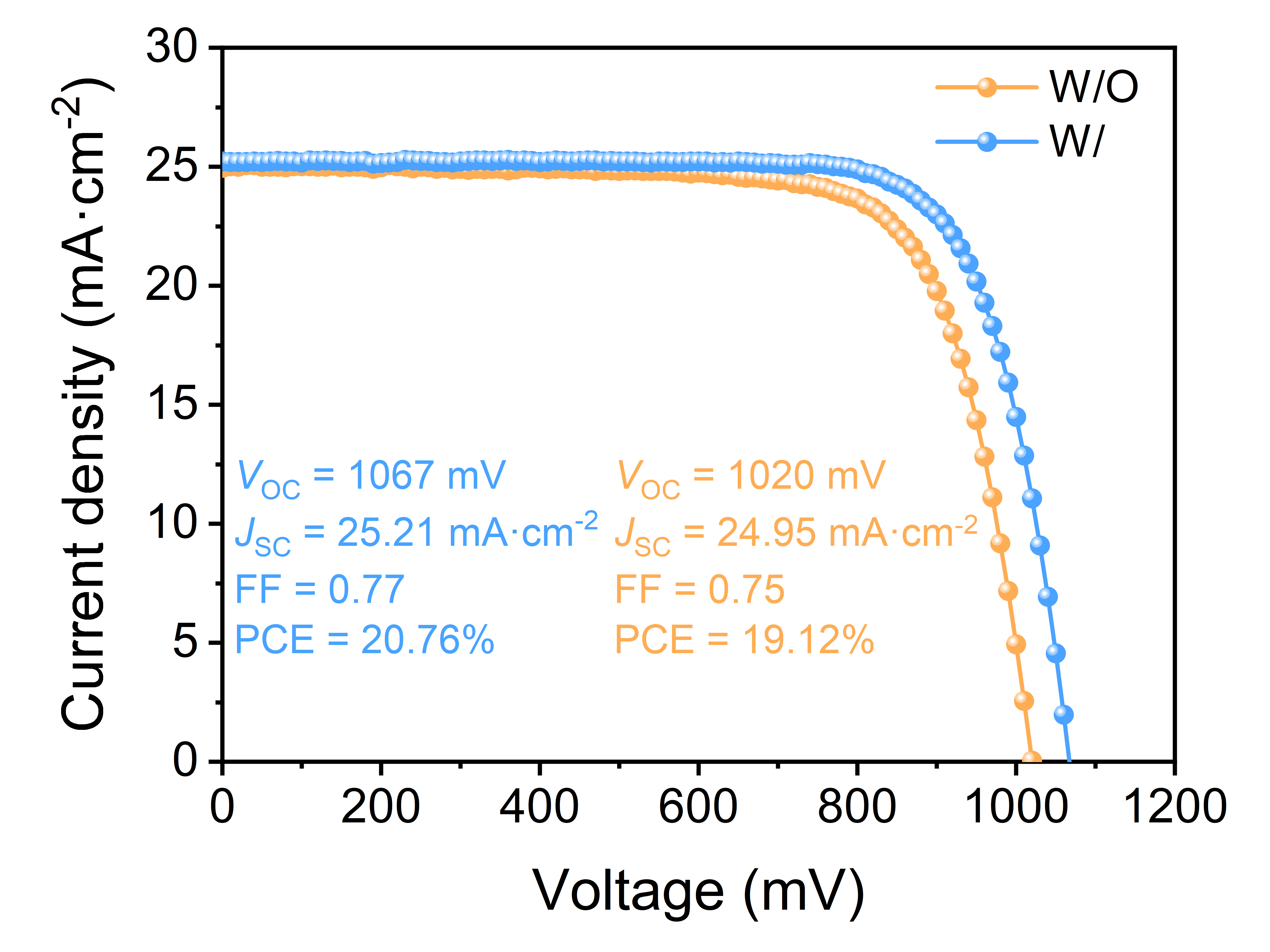


**Fig. S15** Forward *J*-*V* scans of p-MPSCs without and with PCPA

**Table S5** Detailed parameters of forward scan and reverse scan for devices with and without PCPA

|  | **scan** | ***V*_OC_ (mV)** | ***J*_SC_ (mA cm^-2^)** | **FF** | **PCE (%)** | **HI (%)** |
| --- | --- | --- | --- | --- | --- | --- |
| **control** | reverse | 1019 | 25.12 | 0.78 | 20.05 | 4.64 |
|  | forward | 1020 | 24.95 | 0.75 | 19.12 |  |
| **with PCPA** | reverse | 1061 | 25.26 | 0.81 | 21.63 | 4.02 |
|  | forward | 1067 | 25.21 | 0.77 | 20.76 |  |

The HI is calculated as:

$$HI=\frac{{PCE}_{Reverse}-{PCE}_{Forward}}{{PCE}_{Reverse}}$$

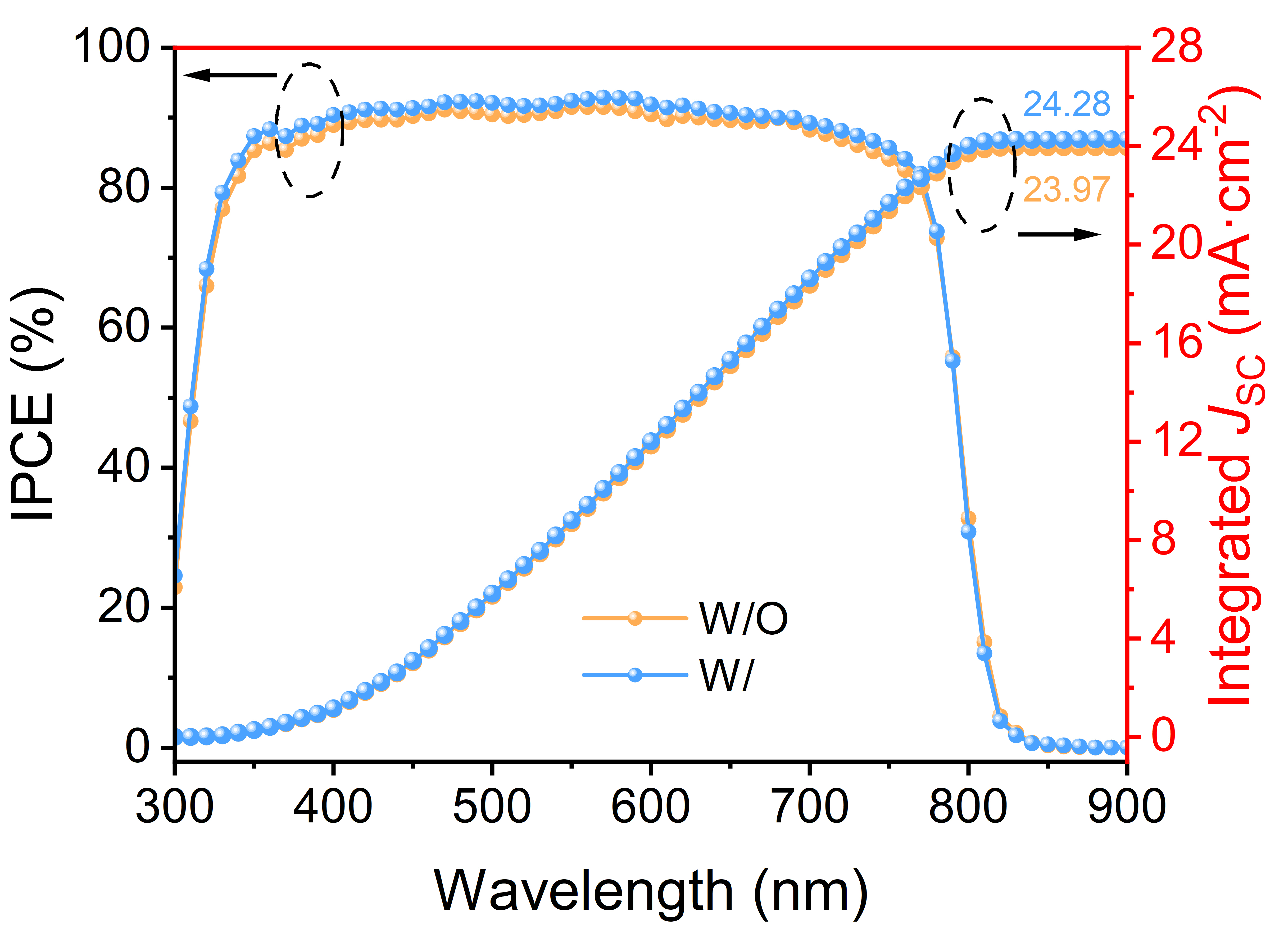


**Fig. S16** IPCE spectra of p-MPSCs without and with PCPA

**Table S6** Initial and final PCEs of p-MPSCs without and with PCPA for the stabilized power output

|  | initial PCE | final PCE |
| --- | --- | --- |
| W/O | 19.71% | 19.71% |
| W/ | 21.62% | 21.51% |

**Table S7** Comparative PCEs of p-MPSCs and PSCs featuring planar, low-temperature-processed carbon electrodes

| **Device structure** | **Highlight** | **Year** | **PCE** | **Refs.** |
| --- | --- | --- | --- | --- |
| High temperature processed HTL-free mesoporous perovskite solar cells | | | | |
| FTO/c-TiO_2_/mp-TiO_2_/mp-ZrO_2_/carbon | Apply polymer additive for defect passivation | 2023 | 18.33% | [S5] |
| FTO/c-TiO_2_/mp-TiO_2_/mp-ZrO_2_/carbon | Mange oxygen vacancies in ETL | 2023 | 18.96% | [S6] |
| FTO/c-TiO_2_/mp-TiO_2_/mp-ZrO_2_/carbon | Optimize electron extraction and transport in ETL | 2024 | 22.2% | [S7] |
| FTO/c-TiO_2_/mp-TiO_2_/mp-ZrO_2_/carbon | Optimize wettability of perovskite solution to the mesoporous films | 2025 | 20.27% | [S8] |
| FTO/c-TiO_2_/mp-TiO_2_/mp-ZrO_2_/carbon | Dual back surface fields for hole transport | 2025 | 20.79% | [S9] |
| Low-temperature processed planar perovskite solar cells | | | | |
| ITO/SnO_2_/PVSK/bi-layer HTL/carbon | Employ bi-layer HTL for optimizing charge carrier loss | 2023 | 19.2% | [S10] |
| ITO/SnO_2_/PVSK/P3HT-NiO_X_/carbon | Construct the protective buffer layers for preventing corrosion | 2023 | 20.14% | [S11] |
| FTO/SnO_2_/PVSK/ carbon | Construct 2D/3D p-n heterojunction to regulate energy level alignment | 2024 | 20.08% | [S12] |
| FTO/SnO_2_/PVSK/ carbon | Construct Pb/Sn-based perovskite heterojunction | 2024 | 19.89% | [S13] |
| ITO/SAM-NiO_X_/PVSK/C_60_/SnO_X_/carbon | First demonstrate carbon as an electron-collecting rear cathode | 2025 | 21.8% | [S14] |
| FTO/SnO2/PVSK/Spiro-OMeTD/carbon | Employ graphene oxide doped HTL to enhance charge extraction | 2025 | 23.6% | [S15] |

**Supplementary References**

1. S. Karthick, S. Velumani, J. Bouclé, Experimental and SCAPS simulated formamidinium perovskite solar cells: a comparison of device performance. Sol. Energy **205**, 349–357 (2020). <https://doi.org/10.1016/j.solener.2020.05.041>
2. Z. Ni, C. Bao, Y. Liu, Q. Jiang, W.-Q. Wu et al., Resolving spatial and energetic distributions of trap states in metal halide perovskite solar cells. Science **367**(6484), 1352–1358 (2020). <https://doi.org/10.1126/science.aba0893>
3. S. Taheri, A. Ahmadkhan kordbacheh, M. Minbashi, A. Hajjiah, Effect of defects on high efficient perovskite solar cells. Opt. Mater. **111**, 110601 (2021). <https://doi.org/10.1016/j.optmat.2020.110601>
4. Y. Zhang, Z. Yang, T. Ma, Z. Ai, C. Wang et al., A theoretical investigation of transport layer-free homojunction perovskite solar cells *via* a detailed photoelectric simulation. Adv. Energy Mater. **13**(12), 2203366 (2023). <https://doi.org/10.1002/aenm.202203366>
5. Z. Zheng, M. Xia, X. Chen, X. Xiao, J. Gong et al., Enhancing the performance of fa-based printable mesoscopic perovskite solar cells *via* the polymer additive. Adv. Energy Mater. **13**(23), 2204335 (2023). <https://doi.org/10.1002/aenm.202204335>
6. J. Liu, S. Li, Z. Qiu, Y. Liu, C. Qiu et al., Stratified oxygen vacancies enhance the performance of mesoporous TiO_2_ electron transport layer in printable perovskite solar cells. Small **19**(32), e2300737 (2023). <https://doi.org/10.1002/smll.202300737>
7. J. Liu, X. Chen, K. Chen, W. Tian, Y. Sheng et al., Electron injection and defect passivation for high-efficiency mesoporous perovskite solar cells. Science **383**(6688), 1198–1204 (2024). <https://doi.org/10.1126/science.adk9089>
8. Y. Cheng, J. Xiang, X. Li, G. Zhang, M. Xia et al., Wettability sequence optimization and interface strain buffering in triple mesoporous layer-based printable perovskite solar cells for enhanced performance. Adv. Mater. **37**(20), 2413151 (2025). <https://doi.org/10.1002/adma.202413151>
9. C. Wang, J. Xiang, J. Liu, C. Han, Z. Zheng et al., Bifunctional compound induced dual back surface fields for efficient hole transport layer-free perovskite solar cells. Adv. Mater. **37**(27), 2502724 (2025). <https://doi.org/10.1002/adma.202502724>
10. T. Du, S. Qiu, X. Zhou, V.M. Le Corre, M. Wu et al., Efficient, stable, and fully printed carbon-electrode perovskite solar cells enabled by hole-transporting bilayers. Joule **7**(8), 1920–1937 (2023). <https://doi.org/10.1016/j.joule.2023.06.005>
11. Y. Li, X. Lu, Y. Mei, C. Dong, D.T. Gangadharan et al., Blade-coated carbon electrode perovskite solar cells to exceed 20% efficiency through protective buffer layers. Adv. Funct. Mater. **33**(34), 2301920 (2023). <https://doi.org/10.1002/adfm.202301920>
12. Y. Lin, J. Tang, H. Yan, J. Lin, W. Wang et al., Ultra-large dipole moment organic cations derived 3D/2D p–n heterojunction for high-efficiency carbon-based perovskite solar cells. Energy Environ. Sci. **17**(13), 4692–4702 (2024). <https://doi.org/10.1039/D4EE00568F>
13. L. Li, Z. Wu, J. Hong, H. Rao, X. Zhong et al., Pb/Sn-based perovskite heterojunction for hole transport layer-free carbon-based perovskite solar cells. ACS Energy Lett. **9**(9), 4240–4247 (2024). <https://doi.org/10.1021/acsenergylett.4c00961>
14. T. Du, H.U. Dag, Z. Peng, J. Englhard, A. Barabash et al., Enhancing the viability of p-i-n perovskite solar cells with printable carbon cathode: Origin of polarity inversion. Joule **10**(1), 102224 (2026). <https://doi.org/10.1016/j.joule.2025.102224>
15. Y. Wang, W. Li, X. Wu, G. Meng, Q. Liu et al., Graphene oxide doping of the hole injection layer enables 23.6% efficiency in perovskite solar cells with carbon electrodes. Nat. Energy (2025). <https://doi.org/10.1038/s41560-025-01893-8>
